# Maternal dietary imbalance between omega-6 and omega-3 fatty acids triggers the offspring’s overeating in mice

**DOI:** 10.1038/s42003-020-01209-4

**Published:** 2020-08-28

**Authors:** Nobuyuki Sakayori, Masanori Katakura, Kei Hamazaki, Oki Higuchi, Kazuki Fujii, Ryoji Fukabori, Yoshio Iguchi, Susumu Setogawa, Keizo Takao, Teruo Miyazawa, Makoto Arita, Kazuto Kobayashi

**Affiliations:** 1 Department of Molecular Genetics, Institute of Biomedical Sciences, Fukushima Medical University, Fukushima, 960-1295 Japan; 2 Japan Society for the Promotion of Science, Chiyoda-ku, Tokyo 102-0083 Japan; 3 Department of Physiology and Oral Physiology, Graduate School of Biomedical and Health Sciences, Hiroshima University, Hiroshima, 734-8553 Japan; 4 Laboratory of Nutritional Physiology, Department of Pharmaceutical Sciences, Faculty of Pharmacy and Pharmaceutical Sciences, Josai University, Sakado, Saitama 350-0295 Japan; 5 Department of Public Health, Faculty of Medicine, University of Toyama, Sugitani, Toyama 930-0194 Japan; 6 New Industry Creation Hatchery Center, Tohoku University, Sendai, Miyagi 980-8579 Japan; 7 Biodynamic Plant Institute Co., Ltd., Sapporo, Hokkaido 001-0021 Japan; 8 Department of Behavioral Physiology, Graduate School of Innovative Life Science, University of Toyama, Sugitani, Toyama 930-0194 Japan; 9 Life Science Research Center, University of Toyama, Sugitani, Toyama 930-0194 Japan; 10 Division for Memory and Cognitive Function, Research Center for Advanced Medical Science, Comprehensive Research Facilities for Advanced Medical Science, Dokkyo Medical University, Mibu-machi, Tochigi 321-0293 Japan; 11 Laboratory for Metabolomics, RIKEN Center for Integrative Medical Sciences, Yokohama, Kanagawa 230-0045 Japan; 12 Graduate School of Medical Life Science, Yokohama City University, Yokohama, Kanagawa 230-0045 Japan; 13 Division of Physiological Chemistry and Metabolism, Keio University Faculty of Pharmacy, Minato-ku, Tokyo 105-0011 Japan

**Keywords:** Neurogenesis, Agriculture, Feeding behaviour, Motivation

## Abstract

The increasing prevalence of obesity and its effects on our society warrant intensifying basic animal research for understanding why habitual intake of highly palatable foods has increased due to recent global environmental changes. Here, we report that pregnant mice that consume a diet high in omega-6 (*n*-6) polyunsaturated fatty acids (PUFAs) and low in omega-3 (*n*-3) PUFAs (an *n*-6^high^/*n*-3^low^ diet), whose *n*-6/*n*-3 ratio is approximately 120, induces hedonic consumption in the offspring by upregulating the midbrain dopaminergic system. We found that exposure to the *n*-6^high^/*n*-3^low^ diet specifically increases the consumption of palatable foods via increased mesolimbic dopamine release. In addition, neurodevelopmental analyses revealed that this induced hedonic consumption is programmed during embryogenesis, as dopaminergic neurogenesis is increased during in utero access to the *n*-6^high^/*n*-3^low^ diet. Our findings reveal that maternal consumption of PUFAs can have long-lasting effects on the offspring’s pattern for consuming highly palatable foods.

## Abstract (web summary)

Sakayori et al. show that feeding pregnant mice with a diet high in omega-6 polyunsaturated fatty acids (PUFAs) and low in omega-3 PUFAs triggers hedonic consumption in the offspring by increasing its dopaminergic neurogenesis. This study suggests that maternal consumption of diets with unbalanced PUFAs contributes to the offspring’s overconsumption of foods.

## Introduction

In nature, consuming a diet high in sugar and fat confers a clear advantage to the organism, as such foods provide readily accessible energy. The instinctive drive to consume such a diet, which has been acquired through evolution^1^, has become an overshoot in our modern society in which highly palatable foods (i.e., hyper-caloric foods) abound. Frequent consumption of fast foods, which are often rich in calories but poor in essential nutrients, has been linked to a variety of health hazards including obesity^2^. Given that no nation has yet succeeded in reducing the rising prevalence of obesity population during the past three decades^3^, identifying the components that contribute to unhealthy eating habits is extremely urgent and has high societal relevance.

The hedonic aspects of ingestive behavior are potentiated by mesolimbic dopaminergic neurons in the ventral tegmental area (VTA) in the midbrain, which innervate the nucleus accumbens (NAc) in the basal ganglia^4,5^. In animal models, the consumption of palatable foods increases extracellular dopamine (DA) levels in the medial NAc^6–8^, and pharmacologically inducing DA release in this brain region increases sucrose consumption^8–10^. Interestingly, reward-predicting cues also activate mesolimbic dopaminergic neurons^11,12^. Moreover, increasing extracellular DA in the medial NAc, which is more responsive to reward-predicting cues compared to the lateral NAc^13^, increases the response to obtaining a predicted sucrose reward^14^. On the other hand, increasing DA release in the medial NAc does not affect water consumption^8^, and DA in the NAc does not play a role in the consumption of a standard (i.e., healthy) diet^15^. Taken together, these data support the notion that inducing DA release in the medial NAc selectively potentiates the consumption of highly palatable foods.

*n*-6 and *n*-3 polyunsaturated fatty acids (PUFAs) are essential components to build cellular structures. Despite being enriched fatty acids in the brain, mammals cannot synthesize these PUFAs de novo and must therefore obtain these nutrients from dietary sources to maintain sufficient and appropriate levels of PUFAs in the brain^16,17^. The fact that these PUFAs compete for incorporation into the cell membrane gives rise to an *n*-6/*n*-3 ratio in the brain. Importantly, modern agriculture and agribusiness have led to foods that are high in *n*-6 PUFAs and low in *n*-3 PUFAs^18,19^. In a recent animal study, we found that consumption of such an imbalanced diet by the pregnant mother increases the *n*-6/*n*-3 ratio in the offspring’s brain and impairs neocortical development^20^. Increase in the *n*-6/*n*-3 ratio in the brain resulting from dietary or genetic alterations is also associated with cognitive and emotional deficits^21–25^. These data underscore the effect that a dietary imbalance between *n*-6 and *n*-3 PUFAs and a consequent increase in the brain *n*-6/*n*-3 ratio can have on brain development and brain function.

Further evidence has emerged showing that the *n*-6/*n*-3 ratio in the membrane of erythrocytes is correlated with weight gain in humans^26^. Taking this evidence into consideration, we hypothesized that consuming a diet high in *n*-6 PUFAs and low in *n*-3 PUFAs (i.e., an *n*-6^high^/*n*-3^low^ diet) can disrupt healthy eating habits. Using nutritionally customized diets, we found that consumption of an *n*-6^high^/*n*-3^low^ diet by pregnant female mice elevates the *n*-6/*n*-3 ratio in the offspring’s brain and leads to increased consumption of highly palatable foods by upregulating mesolimbic dopaminergic system in the offspring.

## Results

### Effect of dietary PUFAs on growth and brain lipids

**Fig. 1:**
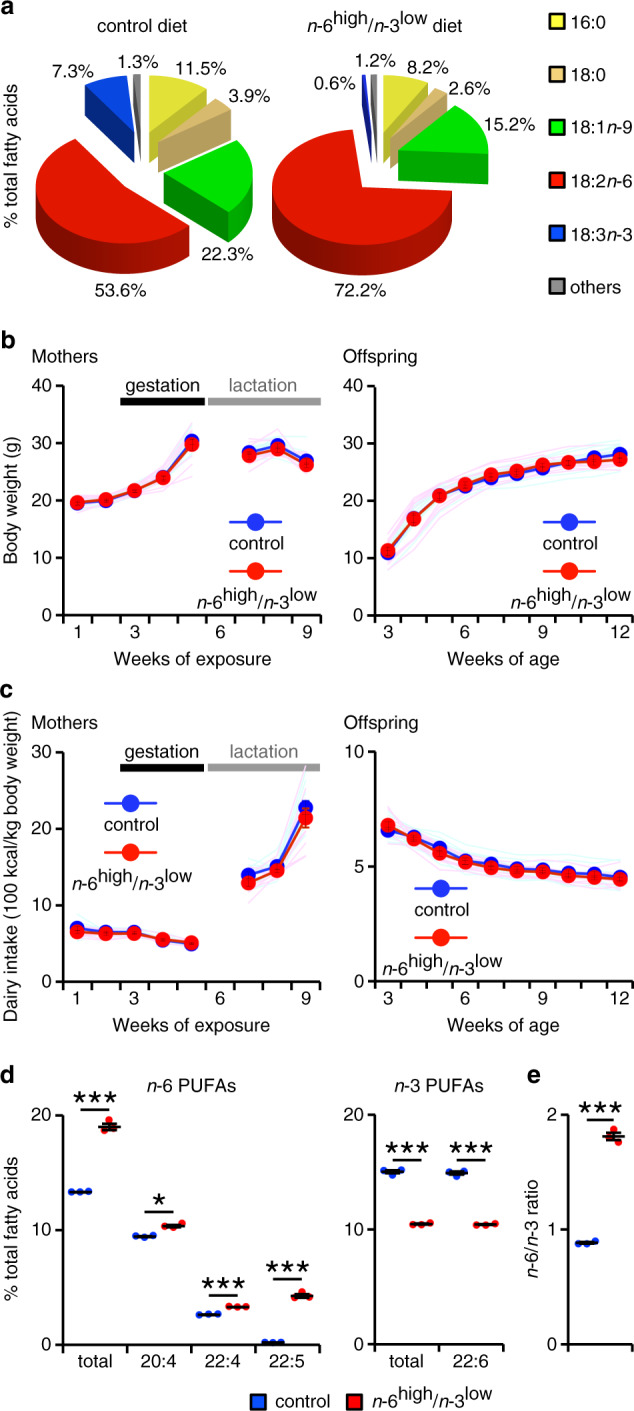
Exposure to the *n*-6^high^/*n*-3^low^ diet increases the *n*-6/*n*-3 ratio in the offspring’s brain without causing obesity. **a** Composition of fatty acids in the control and *n*-6^high^/*n*-3^low^ diets (*n* = 3/diet). **b** Body weight measured in the mothers and offspring in the control and *n*-6^high^/*n*-3^low^ groups (*n* = 10/group). Data were analyzed using a two-way ANOVA (week as repeated measure). **c** Daily food intake measured for the mothers and offspring in the control and *n*-6^high^/*n*-3^low^ groups (*n* = 10/group). Data were analyzed using a two-way ANOVA (week as repeated measure). **d** Levels of *n*-6 and *n*-3 PUFAs in the offspring’s brain in the control and *n*-6^high^/*n*-3^low^ groups (*n* = 3/group). *P < 2.08 × 10^–3^ and ***P < 4.17 × 10^−5^, unpaired Student’s *t* test. **e** The *n*-6/*n*-3 ratio in the offspring’s brain in the control and *n*-6^high^/*n*-3^low^ groups (*n* = 3/group). ***P < 4.17 × 10^−5^, unpaired Student’s *t* test. Data are expressed as the mean (**a**).

To examine the effect of prenatal exposure and postnatal consumption of a diet high in *n*-6 PUFAs and low in *n*-3 PUFAs (an *n*-6^high^/*n*-3^low^ diet), we fed female mice either the control diet or the *n*-6^high^/*n*-3^low^ diet beginning two weeks before mating; this diet was then continued throughout pregnancy and lactation, and the offspring were continued on the same diet after weaning at 3 weeks of age (the male offspring were used for the following experiments, unless otherwise stated). The composition of ingredients was similar between the two diets (Supplementary Fig. 1a), as were the caloric content (Supplementary Fig. 1b, c) and total fatty acids (Supplementary Fig. 1d); however, the composition of *n*-6 and *n*-3 PUFAs, especially linoleic acid (18:2*n*-6) and α-linolenic acid (18:3*n*-3), differed between the two diets, as shown in Fig. 1a, and the *n*-6/*n*-3 ratio in the control and *n*-6^high^/*n*-3^low^ diets was 7.4 ± 0.5 and 121.4 ± 1.9, respectively. Moreover, the peroxide levels in the diets were low for both diets throughout the experiments (Supplementary Fig. 1e). The body weight of both the mothers (during pregnancy and lactation) and the offspring (starting at 3 weeks of age) were similar between the two dietary groups (Fig. 1b); in addition, food intake was similar between the two groups (Fig. 1c). Thus, consuming an *n*-6^high^/*n*-3^low^ diet per se does not affect growth or induce obesity, consistent with previous reports^20,27,28^.

We verified that levels of *n*-6 and *n*-3 PUFAs in the adult offspring’s brain were increased and decreased, respectively, in the *n*-6^high^/*n*-3^low^ group compared to those in the control group (Fig. 1d and Supplementary Fig. 2c, d). Note that the brain *n*-6/*n*-3 ratio in the *n*-6^high^/*n*-3^low^ group was 1.81 ± 0.03 (Fig. 1e) and was within a range of the *n*-6/*n*-3 ratio observed in the postmortem brain of Americans (Supplementary Fig. 2e)^29^. Levels of saturated or monounsaturated fatty acids were not affected in the *n*-6^high^/*n*-3^low^ group (Supplementary Fig. 2a, b). Thus, consuming an *n*-6^high^/*n*-3^low^ diet increases the *n*-6/*n*-3 ratio in the offspring’s brain, as we have reported previously^20^.

Importantly, we also found that the birth rate, litter size, and weaning rate were similar between the two groups (Supplementary Fig. 3). Thus, consuming an *n*-6^high^/*n*-3^low^ diet does not significantly affect such gestational or lactational outcomes.

### Dietary imbalance of PUFAs induces hedonic consumption

**Fig. 2:**
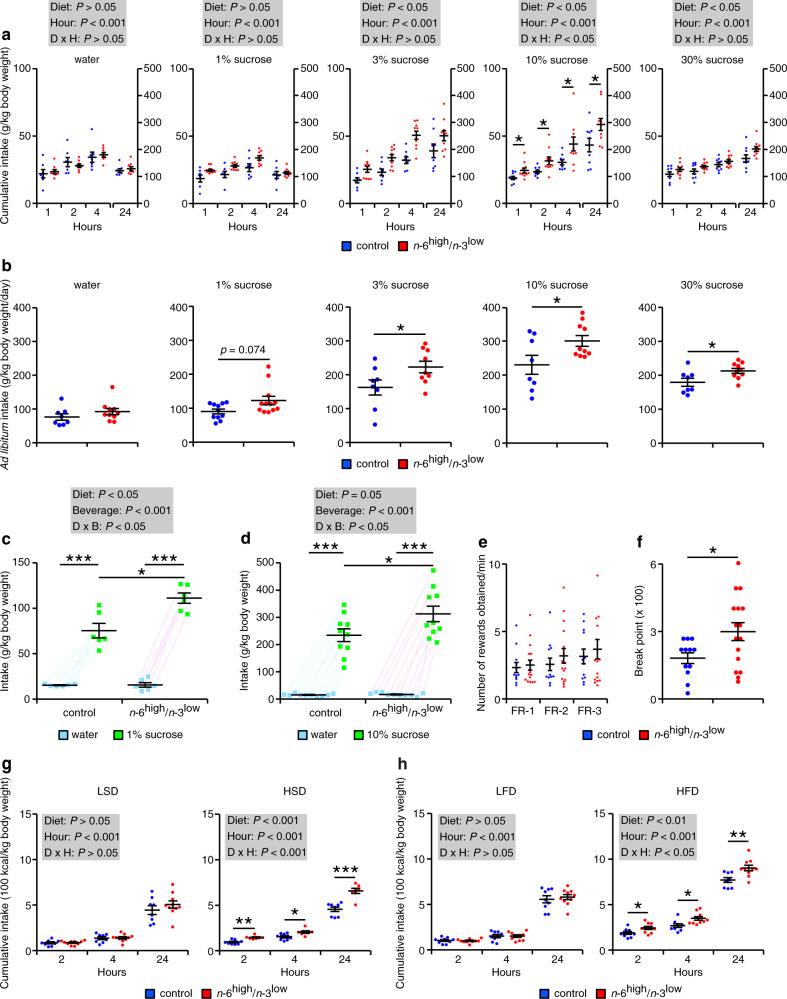
Exposure to the *n*-6^high^/*n*-3^low^ diet increases the consumption of highly palatable foods. **a** Cumulative intake of water, 1%, 3%, 10%, or 30% sucrose solution measured in the control and *n*-6^high^/*n*-3^low^ groups after water deprivation for 12 h (water, *n* = 7/control or 8/*n*-6^high^/*n*-3^low^; 1%, *n* = 7/control or 9/*n*-6^high^/*n*-3^low^; 3%, *n* = 8/control or 10/*n*-6^high^/*n*-3^low^; 10% and 30%, *n* = 9/control or 10/*n*-6^high^/*n*-3^low^). *P < 0.05, two-way ANOVA with simple main effect analysis (hour as repeated measure). **b** Intake of water, 1%, 3%, 10%, or 30% sucrose solution measured in the control and *n*-6^high^/*n*-3^low^ groups without water deprivation (water, 10%, and 30%, *n* = 8/control or 10/*n*-6^high^/*n*-3^low^; 1%, *n* = 11/control or 12/*n*-6^high^/*n*-3^low^; 3%, *n* = 8/control or 9/*n*-6^high^/*n*-3^low^). *P < 0.05, Wilcoxon’s rank sum test (for water and 1% and 10% sucrose solutions) or unpaired Student’s *t*-test (for 3% and 30% sucrose solutions). **c**, **d** Mice in the *n*-6^high^/*n*-3^low^ group consume more sucrose solution (1% and 10%) than those in the control group when given access to both water and sucrose solution (**c**, *n* = 6/group; **d**, *n* = 10/group). *P < 0.05 and ***P < 0.001, two-way ANOVA with simple main effect analysis (consumption of water and sucrose solution as repeated measure). **e** The number of rewards obtained during the training sessions of the PR task (*n* = 12/control or 16/*n*-6^high^/*n*-3^low^). Data were analyzed using a Wilcoxon’s rank sum test. **f** The break point in the PR task (*n* = 12/control or 16/*n*-6^high^/*n*-3^low^). *P < 0.05, Welch’s *t*-test. **g**, **h** Cumulative intake of the indicated diets in the control and *n*-6^high^/*n*-3^low^ groups. *P < 0.05, **P < 0.01, and ***P < 0.001, two-way ANOVA with simple main effect analysis (hour as repeated measure). LSD, *n* = 8/control or 10/*n*-6^high^/*n*-3^low^; HSD, *n* = 8/control or 7/*n*-6^high^/*n*-3^low^; LFD, *n* = 9/control or 10/*n*-6^high^/*n*-3^low^; HFD, *n* = 8/control or 10/*n*-6^high^/*n*-3^low^.

Given the present global situation in which fast foods are more readily available than ever before^2,30^, we examined whether consuming the *n*-6^high^/*n*-3^low^ diet induces hedonic consumption by measuring the intake of water and sucrose-containing solutions in the offspring that were water-deprived for 12 h. We found that although the mice in the *n*-6^high^/*n*-3^low^ group consumed the same amount of water and 1% sucrose solution compared to the mice in the control group, the mice in the *n*-6^high^/*n*-3^low^ group consumed significantly more of the 3%, 10%, and 30% sucrose solutions (Fig. 2a); similar results were obtained when the solutions were available ad libitum (Fig. 2b). This increased consumption of sucrose-containing solution by the mice in the *n*-6^high^/*n*-3^low^ group was also observed when 1% or 10% sucrose solution was provided simultaneously with water, even though the two groups consumed similar amounts of water (Fig. 2c, d). Taken together, these data indicate that mice that consumed an *n*-6^high^/*n*-3^low^ diet since conception develop a stronger preference for sucrose-containing solutions compared to mice that consumed a control diet.

Next, we examined the behavioral consequences of consuming the *n*-6^high^/*n*-3^low^ diet by measuring the offspring’s effort-related motivation to obtain 10% sucrose solution using the progressive ratio (PR) task, which measures the animal’s willingness to work in order to obtain a predicted reward^31^ (see “Methods”). We found no difference between the two groups during the training sessions that were based on fixed ratio reinforcement schedules (Fig. 2e and Supplementary Fig. 4a–d), indicating that the mice in the *n*-6^high^/*n*-3^low^ group have intact associative learning and are physically capable of pressing the lever. We then performed the PR task and found a significant increase in the number of lever presses that mice achieved during the task, which is indicated as break point, in the *n*-6^high^/*n*-3^low^ group compared to the control group (Fig. 2f and Supplementary Fig. 4e); in contrast, we found no difference with respect to pressing the inactive lever (Supplementary Fig. 4f) or the latency between presentation of the reward and accessing the reward (Supplementary Fig. 4g). These results indicate that mice exposed to an *n*-6^high^/*n*-3^low^ diet have an increased motivational drive for obtaining a sucrose-containing solution.

Consistent with their increased consumption of sucrose solutions, the mice in the *n*-6^high^/*n*-3^low^ group gained more weight and consumed more 30% sucrose solution over a 2-week period compared to the mice in the control group (Supplementary Fig. 5d, e), although daily food intake was similar between the two groups (Supplementary Fig. 5f). In contrast, body weight and both water and food intake were similar between the two groups when the mice were given water instead of sucrose solution (Supplementary Fig. 5a–c). Taken together, these data indicate that long-term consumption of an *n*-6^high^/*n*-3^low^ diet induces hedonic behavior, which leads to an overweight state.

Next, we examined whether the increased consumption of palatable foods in the *n*-6^high^/*n*-3^low^ offspring is specific to sucrose solutions by measuring the consumption of a diet containing high amounts of either sucrose (Supplementary Fig. 6a–c) or fat (Supplementary Fig. 6d–f). We found that following food deprivation, the mice in the *n*-6^high^/*n*-3^low^ group consumed significantly more high-sucrose diet (HSD; Fig. 2g) and high-fat diet (HFD; Fig. 2h) compared to the mice in the control group; in contrast, no difference between the two groups was observed when the mice were presented with a low-sucrose diet (LSD) or low-fat diet (LFD) following food deprivation (Fig. 2g, h). Thus, long-term consumption of an *n*-6^high^/*n*-3^low^ diet causes an increase in the intake of both high-sugar and high-fat foods.

Importantly, the differences observed between the two groups were not confounded by locomotor activity or anxious behaviors, as we found no difference in distance traveled (Supplementary Fig. 7a), duration in a center zone (Supplementary Fig. 7b), or entries into the center zone (Supplementary Fig. 7c) in the open field test. Moreover, when water was presented as the reward in the training sessions for the PR task, pressing the lever was not reinforced (Supplementary Fig. 4b), indicating that pressing the lever was indeed reinforced specifically by the sucrose solution reward.

### Dietary imbalance of PUFAs potentiates DA release

**Fig. 3:**
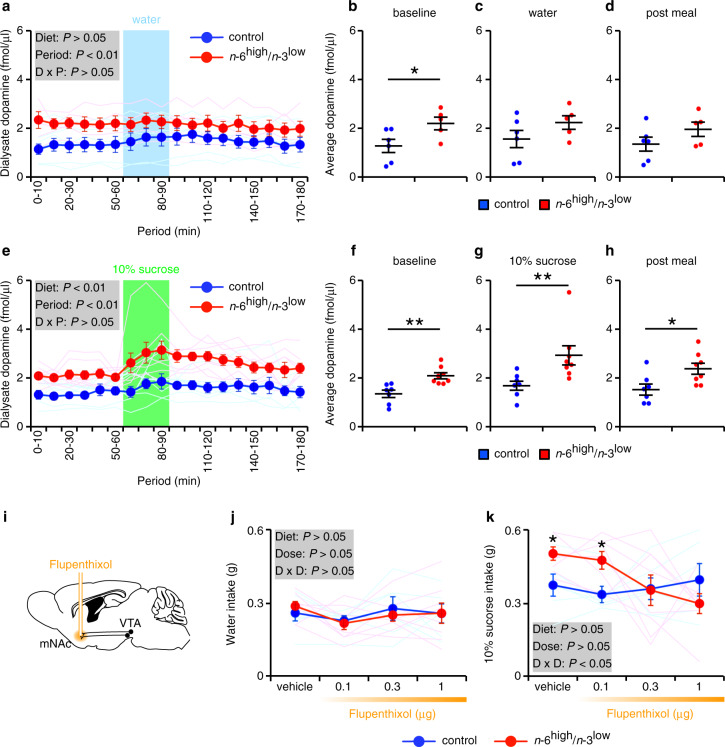
Exposure to the *n*-6^high^/*n*-3^low^ diet increases DA release in the medial NAc. **a**, **e** Time course of dialysate DA concentration measured in the medial NAc (mNAc); where indicated, water or 10% sucrose was provided. Data were analyzed using a two-way ANOVA (period as repeated measure). **b**–**d**, **f**–**h** Dialysate DA concentration measured in the mNAc before, during, and after access to either water or 10% sucrose. *P < 0.05 and **P < 0.01, unpaired Student’s *t* test (**b**–**d**, **f**, **h**) or Wilcoxon’s rank sum test (**g**). **a**–**d**, *n* = 6/control or 5/*n*-6^high^/*n*-3^low^; **e**–**h**, *n* = 7/control or 8/*n*-6^high^/*n*-3^low^. **i** Schematic diagram depicting the infusion of flupenthixol in the mNAc; the location of the VTA is also indicated. **j**, **k** The intake of water or 10% sucrose solution measured in mice following an infusion of vehicle or the indicated dose of flupenthixol in each hemisphere (*n* = 6/control or 8/*n*-6^high^/*n*-3^low^). *P < 0.05, two-way ANOVA with simple main effect analysis (dose as repeated measure).

To investigate the neuronal mechanism that underlies hedonic consumption in the *n*-6^high^/*n*-3^low^ group, we used in vivo microdialysis to measure DA levels in the medial NAc before, during, and after access to either water or 10% sucrose in mice that were water-deprived for 12 h (Fig. 3a, e and Supplementary Fig. 8). Under baseline conditions, the dialysate DA concentration was significantly higher in the *n*-6^high^/*n*-3^low^ group compared to the control group (Fig. 3b, f). Although dialysate DA was similar between the two groups both during and after access to water (Fig. 3c, d), DA concentration was significantly higher in the *n*-6^high^/*n*-3^low^ group both during and after access to 10% sucrose (Fig. 3g, h). Taken together, these results suggest that increased DA levels in the medial NAc play a role in the hedonic behavior in mice raised on an *n*-6^high^/*n*-3^low^ diet.

Next, we examined whether inhibiting dopaminergic signaling in the medial NAc can reduce hedonic consumption in the *n*-6^high^/*n*-3^low^ group by infusing the non-selective DA receptor antagonist flupenthixol into the medial NAc of water-deprived mice and measuring their consumption of either water or 10% sucrose solution (Fig. 3i and Supplementary Fig. 9). As expected, flupenthixol had no effect on water intake in either group (Fig. 3j); in contrast, infusing either 0.3 or 1 μg flupenthixol/hemisphere reduced sucrose solution intake in the *n*-6^high^/*n*-3^low^ group to the same level as the control group (Fig. 3k), supporting the notion that dopaminergic signaling in the medial NAc plays a critical role in inducing hedonic consumption in the *n*-6^high^/*n*-3^low^ group.

### Dietary imbalance of PUFAs increases dopaminergic neurons

**Fig. 4:**
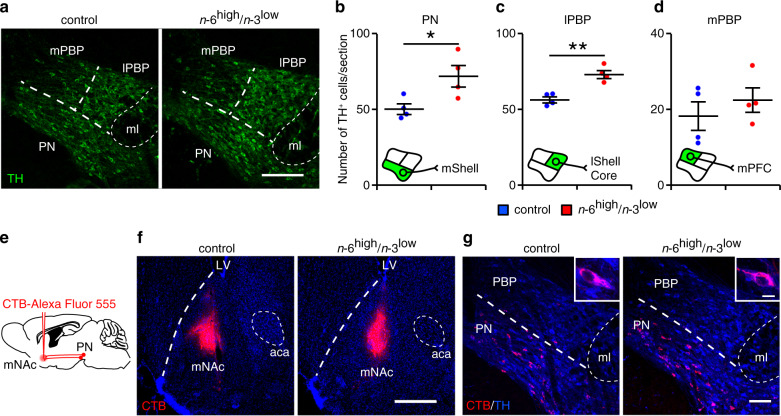
Exposure to the *n*-6^high^/*n*-3^low^ diet increases the number of dopaminergic neurons in the VTA. **a–d** TH-positive dopaminergic neurons measured in the PN, lPBP, and mPBP in the control and *n*-6^high^/*n*-3^low^ groups (*n* = 4/group). *P < 0.05, **P < 0.01, unpaired Student’s *t* test. **e** Schematic diagram depicting the injection of cholera toxin B subunit (CTB)-Alexa Fluor 555 into the medial NAc (mNAc) for retrograde tracing. **f**, **g** Representative images of the mNAc (**f**) and the PN and PBP (**g**) after injecting CTB-Alexa Fluor 555 in the mNAc; the nuclei were counterstained with DAPI (blue, **f**). Scale bars = 200 μm (**a**), 500 μm (**f**), 100 μm (**g**), and 10 μm (**g**, inset). aca, anterior commissure; lShell, lateral shell of the NAc; LV, lateral ventricle; ml, medial lemniscus; mPFC, medial prefrontal cortex; mShell, medial shell of the NAc.

Dietary *n*-6 and *n*-3 PUFAs induce neurogenesis in the brain^32–34^, which could explain the observed increase in dialysate DA concentrations in the *n*-6^high^/*n*-3^low^ group. To test this possibility, we measured the number of tyrosine hydroxylase (TH)-positive dopaminergic neurons in all seven subregions of the VTA^35^. We found significantly more dopaminergic neurons in the paranigral nucleus (PN) and the interfascicular nucleus (IF) in the *n*-6^high^/*n*-3^low^ group compared to the control group (Fig. 4a, b and Supplementary Fig. 10b, d), regions in which dopaminergic neurons innervate the medial shell of the NAc^36–38^. There were more dopaminergic neurons also in the parabrachial pigmented nucleus (PBP) in the *n*-6^high^/*n*-3^low^ group (Supplementary Fig. 10d). Here, we further examined the dopaminergic subpopulation in the PBP, and we found more dopaminergic neurons in the lateral PBP (lPBP) in the *n*-6^high^/*n*-3^low^ group (Fig. 4a, c), from which dopaminergic neurons project to the lateral shell and core of the NAc and induce positive preference behavior^36–38^, but not in the medial PBP (mPBP) (Fig. 4a, d), in which dopaminergic neurons innervate the medial prefrontal cortex and induce aversive behavior^36,37^. In addition, we found no difference between the two groups with respect to the number of dopaminergic neurons in the rostral VTA subregions (Supplementary Fig. 10a, d), which are not involved in the rewarding effects of stimuli^13^, or in the caudal linear nucleus (CLi; Supplementary Fig. 10c, d). As a control, we confirmed that the pattern of dopaminergic projections between the VTA and NAc was not affected in the *n*-6^high^/*n*-3^low^ group (Fig. 4e–g and Supplementary Fig. 10e, f), and that the additional neurons in the *n*-6^high^/*n*-3^low^ group did not express dopamine β-hydroxylase (DBH) (Supplementary Fig. 10g, h), confirming that these TH-positive neurons are not noradrenergic. These results support the notion that consuming an *n*-6^high^/*n*-3^low^ diet increases the production of VTA dopaminergic neurons in the specific subregions, thereby increasing DA release in the medial NAc.

Endocannabinoid (eCB) biosynthesis in the brain has been shown to be induced by an increased brain *n*-6/*n*-3 ratio^39^, and eCB signaling is involved in preference behaviors for highly palatable foods by regulating activity of dopaminergic neurons in the VTA^40^. To investigate whether eCB biosynthesis is affected in the *n*-6^high^/*n*-3^low^ group, we measured the amounts of eCBs and related molecules in the ventral midbrain using liquid chromatography-tandem mass spectrometry (LC/MS/MS). We found that the amounts of these molecules were not different in the control and *n*-6^high^/*n*-3^low^ groups (Supplementary Fig. 11). These results show that the induced hedonic consumption in the *n*-6^high^/*n*-3^low^ group is not attributable to altered eCB biosynthesis.

We further investigated the effect of exposure to the *n*-6^high^/*n*-3^low^ diet on female offspring. We similarly found a similar increase in the number of dopaminergic neurons in the VTA, as well as an increased brain *n*-6/*n*-3 ratio and intact body weight and food intake in the *n*-6^high^/*n*-3^low^ group (Supplementary Fig. 12). Unfortunately, we were unable to obtain any reproducible data on the intake of the 10% sucrose solution in female offspring; this might be because ovarian hormones, which fluctuate within a few days in mice, modulated their mesolimbic DA reward systems^41^. We conclude that consuming an *n*-6^high^/*n*-3^low^ diet has a similar impact on the female offspring, but the behavioral outcomes are partially detectable in the present experimental system.

### Hedonic behavior is induced during a critical dietary period

**Fig. 5:**
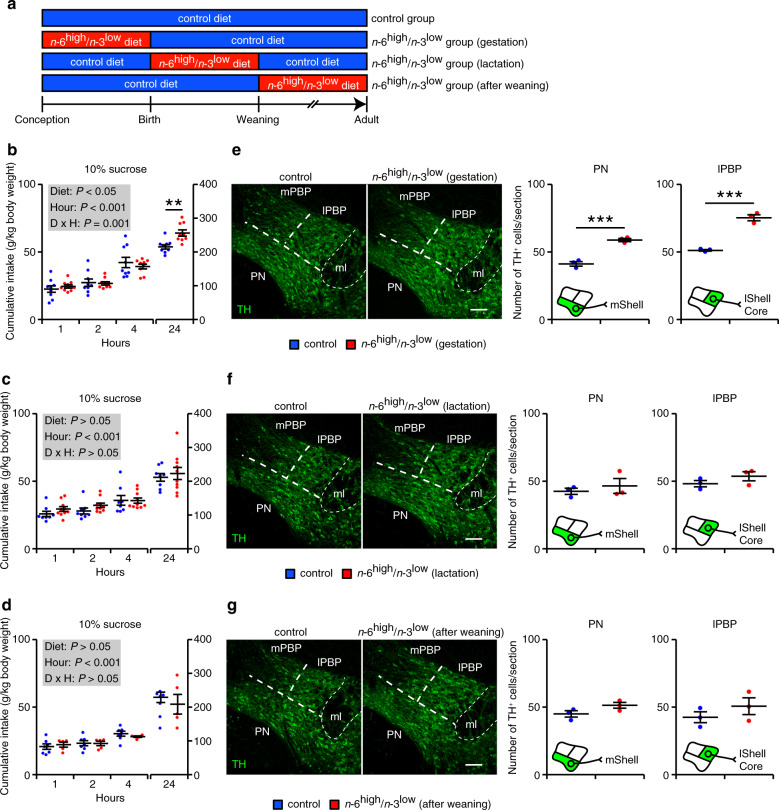
In utero exposure to the *n*-6^high^/*n*-3^low^ diet increases the number of dopaminergic neurons in the VTA and induces hedonic consumption. **a** Experimental outline showing the timing of exposure to either the control diet or the *n*-6^high^/*n*-3^low^ diet. **b**–**g** Cumulative intake of 10% sucrose solution (**b**–**d**) and TH-positive dopaminergic neurons in the PN and lPBP (**e**–**g**) measured in mice in the control group and in mice exposed to the *n*-6^high^/*n*-3^low^ diet during gestation (**b**, **e**), during lactation (**c**, **f**), or after weaning (**d**, **g**) (**b**, *n* = 9/group; **c**, *n* = 8/control or 10/*n*-6^high^/*n*-3^low^; **d**, *n* = 7/control or 5/*n*-6^high^/*n*-3^low^; **e-g**, *n* = 3/group). **P < 0.01 and ***P < 0.001, two-way ANOVA with simple main effect analysis (hour as repeated measure) (**b**–**d**) or unpaired Student’s *t* test (**e**–**g**). Scale bars = 100 μm. lShell, lateral shell of the NAc; ml, medial lemniscus; mShell, medial shell of the NAc.

Given that dopaminergic neurons are produced during embryogenesis, we examined whether hedonic behavior is induced during a specific stage of development. We therefore exposed mice to the *n*-6^high^/*n*-3^low^ diet at three distinct times (before conception through gestation, from birth until weaning, and after weaning) (Fig. 5a) and measured water intake, sucrose intake, and dopaminergic neurons (Fig. 5b–g and Supplementary Fig. 13) in adulthood. We found that the mice that were exposed in utero to the *n*-6^high^/*n*-3^low^ diet had increased intake of the 10% sucrose solution, but not water, compared to mice that were always exposed to the control diet (Fig. 5b and Supplementary Fig. 13a). In contrast, exposure to the control diet in utero followed by the *n*-6^high^/*n*-3^low^ diet at any time after birth had no effect on the intake of either water or 10% sucrose solution (Fig. 5c, d and Supplementary Fig. 13b, c). Consistent with these results, we found that the number of dopaminergic neurons was increased only in the offspring that were exposed to the *n*-6^high^/*n*-3^low^ diet in utero (Fig. 5e–g and Supplementary Fig. 13d–i). These results suggest that the hedonic consumption observed in the adult mice is programmed during embryogenesis in response to the maternal consumption of the *n*-6^high^/*n*-3^low^ diet.

**Fig. 6:**
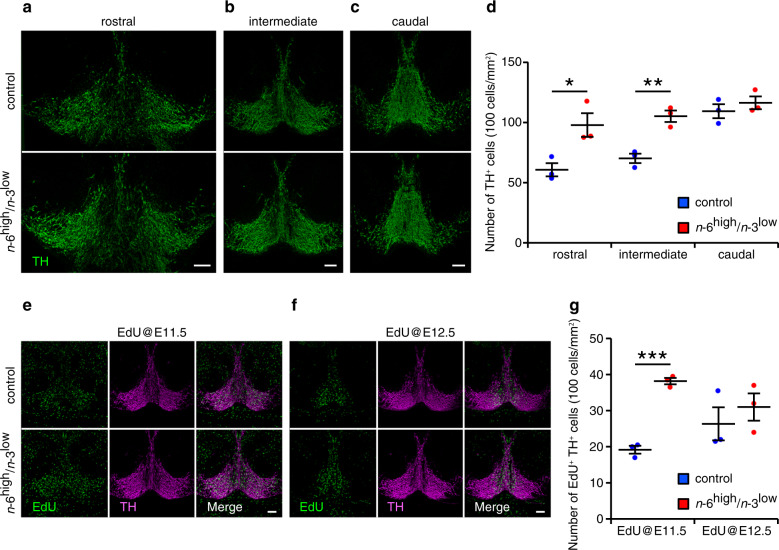
Exposure to the *n*-6^high^/*n*-3^low^ diet induces dopaminergic neurogenesis in the developing midbrain. **a**–**d** TH-positive dopaminergic neurons measured in the rostral, intermediate, and caudal midbrain at E14.5 (*n* = 3/group). *P < 0.05 and **P < 0.01, unpaired Student’s *t* test. **e**–**g**, EdU-labeled TH-positive dopaminergic neurons measured in the intermediate midbrain at E14.5 (*n* = 3/group). ***P < 0.001, unpaired Student’s *t* test. Scale bars = 100 μm.

Lastly, we determined the developmental stage in which dopaminergic neurons are increased in specific subregions of the VTA. We first examined the spatiotemporal pattern of dopaminergic neurogenesis in developing mice under a standard dietary condition. We labeled newborn neurons by intraperitoneal injection of 5-ethynyl-20-deoxyuridine (EdU) in the pregnant mothers at embryonic day 10.5 (E10.5), E11.5, E12.5, E13.5, or E14.5; we then measured the number of EdU-labeled TH-positive dopaminergic neurons in the offspring at postnatal day 7 (P7). We found that neurogenesis in the VTA peaked at E11.5–12.5, depending on the subregion analyzed (Supplementary Fig. 14). Using these data, we then examined dopaminergic neurogenesis in the midbrain of the control and *n*-6^high^/*n*-3^low^ groups at E14.5, the stage at which the majority of dopaminergic neuronal differentiation is completed. Even at this early developmental stage, we observed morphological heterogeneity in TH-positive regions along the rostrocaudal axis (Fig. 6a–c). Importantly, we found that the number of TH-positive dopaminergic neurons was higher in the rostral and intermediate midbrain regions—but not in the caudal region—in the *n*-6^high^/*n*-3^low^ group compared to the control group (Fig. 6d). The increased dopaminergic neurons in the rostral region at E14.5, which was not observed in the adult rostral VTA (see Supplementary Fig. 10a, d), is likely due to an expanded population of dopaminergic neurons in the substantia nigra pars compacta (SNc); this was confirmed by our finding of more TH-positive dopaminergic neurons in the SNc of adult mice in the *n*-6^high^/*n*-3^low^ group (Supplementary Fig. 15). We further evaluated dopaminergic neurogenesis in the intermediate midbrain region by intraperitoneal injection of EdU in the pregnant mothers at E11.5 or E12.5. We found that the number of EdU-labeled TH-positive dopaminergic neurons was increased in the *n*-6^high^/*n*-3^low^ group at E14.5 compared to the control group when newborn neurons were labeled by EdU at E11.5, but not at E12.5 (Fig. 6e–g). Regarding postnatal apoptosis in the midbrain^42^, we found no difference between the groups in the number of active Caspase 3-positive cells in the intermediate midbrain regions (Supplementary Fig. 16). Taken together, these data indicate that exposure to an *n*-6^high^/*n*-3^low^ diet during a specific window of development leads to an increased production of dopaminergic neurons in the midbrain.

## Discussion

The current global epidemic of obesity has occurred in a relatively genetically stable population, suggesting that other factors such as changes in diet underlie the increased consumption of foods that exceed our caloric requirements. The *n*-6/*n*-3 ratio in our diet warrants particular attention, given that this ratio has increased in parallel with the rise in the prevalence of obesity^19^. Here, we used a combination of nutritional, neurobiological, and developmental analyses and revealed that maternal consumption of an *n*-6^high^/*n*-3^low^ diet increases hedonic consumption in the offspring. In addition, we showed that this effect can be attributed to an increase in dopaminergic signaling in the mesolimbic system. To our surprise, this hedonic behavior does not develop in mice that are exposed to the *n*-6^high^/*n*-3^low^ diet exclusively during postnatal development, indicating a relatively narrow window of susceptibility during fetal development.

Exposure to an *n*-6^high^/*n*-3^low^ diet is a potentially obesogenic factor, particularly when coupled with increased availability of highly palatable foods. We found that long-term exposure to the *n*-6^high^/*n*-3^low^ diet (beginning before conception) did not increase the intake of either water or normal food, nor did these mice become overweight; this is consistent with a previous report showing that exposing rats to an *n*-6^high^/*n*-3^low^ diet did not affect body weight^27^. On the other hand, consuming a high-fat diet containing high levels of linoleic acid—an *n*-6 PUFA—from gestational periods through adulthood leads to more severe obesity than consuming a high-fat diet containing low levels of linoleic acid^43^. Moreover, feeding pregnant mice an *n*-3 PUFA-deficient diet during gestation and lactation also increases an instrumental responses reinforced by sucrose tablets in the adult offspring^44^. Here, we found that the increased intake of highly palatable foods in the *n*-6^high^/*n*-3^low^ group was specific to foods that are rich in either sucrose or fat. Moreover, we found that in utero exposure to the *n*-6^high^/*n*-3^low^ diet through the mother’s diet was sufficient to induce hedonic consumption in the offspring. Indeed, the dietary *n*-6/*n*-3 ratio in pregnant women has been associated with the likelihood of developing obesity in their children^45,46^. Thus, an important—yet currently unanswered—question is whether the maternal consumption of an *n*-6^high^/*n*-3^low^ diet increases the child’s risk of developing hedonic eating habits. In this respect, our data could provide a fundamental roadmap for designing epidemiological studies to investigate the putative relationship between maternal PUFA consumption and eating patterns in their children, although any attempts to directly apply our findings to humans should be interpreted with caution.

Total caloric intake plays a clear role in regulating body weight. In lactating mice, maternal consumption of excess fatty acids via a high-fat diet has been associated with the future onset of metabolic disorders in the offspring^47^, indicating that the amount of fatty acids consumed by the mother is an important determinant of the offspring’s future metabolic state. Here, we used isocaloric diets and confirmed that caloric intake was similar between the control and *n*-6^high^/*n*-3^low^ groups during their growth period; however, the adult mice in the *n*-6^high^/*n*-3^low^ group exhibited obesogenic hedonic behavior. Thus, exposure to an *n*-6^high^/*n*-3^low^ diet induces hedonic consumption, even when the diet contains the same total amount of fatty acids as the control diet, providing compelling evidence that the type of fatty acids consumed by the mother is important for preventing obesity in the offspring.

Mesolimbic dopaminergic neurons would facilitate hedonic feeding, but do not cause the basal consumption of palatable foods^4,5^. Interestingly, pharmacologically increasing extracellular DA levels in the medial NAc increases the consumption of sucrose solution^8,10^ and sugar^9^, whereas inhibiting dopaminergic signaling has no effect on the consumption of palatable foods; indeed, even DA-deficient mice have a clear preference for sucrose solution over unsweetened water^48^. Moreover, infusing the non-selective dopamine receptor antagonist flupenthixol into the medial NAc does not affect the consumption of sucrose solution^49^, which is consistent with our data obtained with flupenthixol-treated mice in the control group (see Fig. 3k). These seemingly incongruent behavioral outputs caused by increasing and decreasing mesolimbic dopaminergic signaling are supported by the previous report showing that the increased consumption of sucrose solution due to pharmacologically increasing extracellular DA levels is blocked by concurrently infusing DA receptor antagonists into the medial NAc^8^. We observed a similar “rescue” effect in the *n*-6^high^/*n*-3^low^ group treated with flupenthixol (see Fig. 3k).

Although precisely how DA mediates hedonic consumption is not currently known, it seems likely that a key factor in modulating hedonic feeding in the *n*-6^high^/*n*-3^low^ group is the absolute level of DA, as we found that DA levels in the medial NAc were increased in these mice both before and during access to a 10% sucrose; similar results in baseline DA concentration were reported in rats exposed to an *n*-6^high^/*n*-3^low^ diet^50^. Together with elevated levels of DA, we observed an increase in sucrose solution intake in the *n*-6^high^/*n*-3^low^ group, which was altered by inhibiting DA receptors. A recent report also supports a model that motivated behaviors are regulated by absolute changes in extracellular DA concentrations^51^. It is therefore reasonable to speculate that the absolute concentration of extracellular DA serves to modulate hedonic behavior in the *n*-6^high^/*n*-3^low^ group.

Despite extensive research regarding the anatomical and functional heterogeneity of dopaminergic neurons in the VTA^36–38,52,53^, their pattern of spatiotemporal production in the developing midbrain remains poorly understood. Nevertheless, previous studies showed that dopaminergic neurogenesis in the VTA peaks at E11.5 and E13.5 in mice and rats, respectively^54,55^. We therefore generated a detailed spatiotemporal map of when distinct subpopulations of dopaminergic neurons in the VTA are generated, finding that the majority of dopaminergic neurons are produced at E11.5-E12.5 in mice, the precise timing of which differs among subregions. These data indicate that the heterogeneity of mesolimbic dopaminergic neurons is at least partially determined at the time of neurogenesis, although molecular diversity in dopaminergic neurons is established in later stages^56^. In this respect, our study could serve as a prototype for future neurodevelopmental studies that focus on the spatiotemporal production of dopaminergic neurons in the VTA.

Several human studies found that maternal consumption of an *n*-6^high^/*n*-3^low^ diet can impair the child’s cognitive development^57^, giving rise to the notion that exposure to a well-balanced dietary *n*-6/*n*-3 ratio is essential for normal brain development. Previous animal studies by our group and others provided direct neurodevelopmental evidence that maternal intake of an *n*-6^high^/*n*-3^low^ diet reduces the production of neocortical glutamatergic projection neurons^20,58^. Here, we found that the production of dopaminergic neurons in the midbrain is increased in offspring exposed to an *n*-6^high^/*n*-3^low^ diet, providing compelling evidence that dietary PUFAs differentially affect neurogenesis in the neocortex and midbrain.

There are some possible limitations in this study. First, we used only linoleic acid (18:2*n*-6) and α-linolenic acid (ALA or 18:3*n*-3) as the dietary sources of *n*-6 and *n*-3 PUFAs, respectively. Dietary *n*-6 PUFAs are mainly composed of linoleic acid in humans, but *n*-3 PUFAs in human diets are composed of not only ALA but also eicosapentaenoic acid (20:5*n*-3) and docosahexaenoic acid (DHA or 22:6*n*-3)^59,60^. We used only ALA in the diets because rodents can metabolically convert ALA to DHA. However, a recent study has reported that consumption of a diet containing 6.0% of ALA in total fatty acids decreased the brain levels of DHA in rats compared to those of DHA in rats fed a diet containing 3.7% of ALA in total fatty acids, indicating that dietary ALA might impair DHA synthesis^61^. Considering human situations, differences in the dietary source of *n*-3 PUFAs must be more carefully addressed because humans cannot effectively metabolize ALA to DHA^62^. Use of a diet containing not only ALA but also eicosapentaenoic acid and DHA might establish more sophisticated animal models for translational research. Second, the *n*-6/*n*-3 ratio in the *n*-6^high^/*n*-3^low^ diet (121.4 ± 1.9) is considerably higher than modern human diets, whose *n*-6/*n*-3 ratio is generally within a range of 4–20^19^. It is thus difficult to assert that our diet fully reflects human dietary situations; however, it is worth noting that the brain *n*-6/*n*-3 ratio in the *n*-6^high^/*n*-3^low^ group (1.81 ± 0.03) was within the range of that in the human brain^29^. We conclude that the brain *n*-6/*n*-3 ratio in our animal model is, at least partially, compatible with the human brain state. Third, we evaluated the fatty acid contents in the whole brain and the amounts of eCBs in the ventral midbrain, but not in other specific brain regions. The fatty acid contents and eCB biosynthesis would be altered in many brain regions of the *n*-6^high^/*n*-3^low^ offspring, which might affect hedonic behavior. Fourth, electrophysiological characteristics of dopaminergic neurons in the VTA were not evaluated in this study. We focused on extracellular DA concentrations based on a recent report^63^ proposing that motivated behavior-related DA release in the NAc is independent of firing of VTA dopaminergic neurons and that motivated behaviors are associated with slowly fluctuating DA levels, which can be evaluated by using in vivo microdialysis. Note that correlations between VTA dopaminergic neuronal firing and hedonic consumption have not yet been revealed. The electrophysiological impact of consuming the *n*-6^high^/*n*-3^low^ diet on dopaminergic neurons in the VTA should be investigated in future studies. Taken together, experimental improvements related to those mentioned above are needed for a better understanding of the impacts of consuming an *n*-6^high^/*n*-3^low^ diet.

In the wild, the drive to obtain foods containing high amounts of sugar and/or fat is beneficial for self-preservation and procreation, as it helps ensure both sufficient and efficient sources of energy. In humans, however, consuming an *n*-6^high^/*n*-3^low^ diet can lead to maladaptive feeding practices, particularly in our modern society in which highly palatable foods are abundant. In an attempt to counteract the spread of obesity, a growing number of countries have adopted various health policies such as increasing taxes on unhealthy foods, improving nutrition labeling, and applying regulations to food advertising; despite these efforts, however, the global incidence of obesity has not declined^3^. Our study suggests that the increased availability of *n*-6^high^/*n*-3^low^ diets would hamper the efficacy of these national policies. To create a sustainable society, decreasing the maternal consumption of *n*-6^high^/*n*-3^low^ foods might serve as a preemptive step toward reducing the risks of obesity in future generations. In this respect, our findings may have wide-reaching implications for solving an ongoing global health crisis.

## Methods

### Animals

C57BL/6J mice were obtained from Clea Japan (Tokyo, Japan) and housed at Fukushima Medical University under a standard 12-h light/12-h dark schedule (with the lights on at 7:00 a.m.). Food and water were available ad libitum, unless stated otherwise. E0.5 was defined as midday on the day in which a vaginal plug was observed. Male offspring were used for the experiments, unless stated otherwise. Embryonic and early postnatal male offspring were identified by genotyping as described previously^64^. Offspring derived from at least three independent litters were used for the experiments. All animal experiments were performed in accordance with the National Institutes of Health Guidelines for the Care and Use of Laboratory Animals and were approved by our university’s committee for animal experimentation.

### Diets

Starting at 11 weeks of age, virgin female mice were fed either the control diet or the *n*-6^high^/*n*-3^low^ diet (Supplementary Fig. 1a) manufactured by Clea Japan. Mice were randomly allocated to the dietary groups. The control diet was based on the AIN-93G diet^65^. The *n*-6^high^/*n*-3^low^ diet was produced by replacing the soybean oil in the control diet with the high-linoleic safflower oil, as previously reported^20,28,58^. After 2 weeks of consuming their respective diet, the mice were mated and were maintained on their respective diet through gestation and lactation, unless stated otherwise. After weaning, the offspring were group-housed at 2–4 mice per cage and were fed their respective diet, unless otherwise stated. The diets were stored in the dark at 4 °C with AGELESS oxygen absorber (Mitsubishi Gas Chemical, Tokyo, Japan). The diets exposed to mice were exchanged twice weekly and were newly manufactured at least every three months, as recommended previously^65^. Under these storage conditions, dietary lipids do not undergo peroxidization (Supplementary Fig. 1e).

The composition of the LSD, HSD, LFD, and HFD is shown in Supplementary Fig. 6a, d; these diets were also manufactured by Clea Japan. The composition of LSD and HSD was based on the AIN-93M diet^65^, and the content of sucrose was modified as previously reported^66^. The HFD32 diet and its respective control diet were obtained from Clea Japan and were used as HFD and LFD, respectively.

### Fatty acid analysis of brain lipid fractions

Fatty acid composition in the mouse brain was evaluated using gas chromatography as previously described^67^. Data were collected and analyzed by using the Smart Chrom version 2.27J (Techno Alpha, Tokyo, Japan). The *n*-6/*n*-3 ratio in the human brain was quantified by re-evaluating previously reported data obtained from American postmortem brain samples based on an agreement with the original authors^29^. We focused on these data because they contain levels of docosapentaenoic acid (22:5*n*-6) similar to our animal data.

### Evaluation of dietary lipids

Fatty acid composition and lipid peroxidation in the diets were measured by Japan Food Research Laboratories (Tokyo, Japan). Dietary fatty acids were measured using gas chromatography as previously reported^68^, and the peroxide levels in the diets were measured using the standard chloroform method^69^.

### Liquid and food intake

Each test was initiated at approximately 1 p.m. Before the test, the mice were acclimated to the sucrose solution, LSD, HSD, LFD, HFD, and the bedding paper (Oriental Yeast, Tokyo, Japan) in order to prevent neophobia.

The sucrose consumption test was performed in single-housed mice. Each mouse was offered a drinking bottle containing either tap water or a sucrose solution with or without prior water deprivation for 12 h. After 1, 2, 4, and 24 h, the amount of solution consumed and the animal’s body weight were measured. Intake was calculated as the ratio between the amount of solution consumed (in g) and the animal’s body weight (in kg). During the test, each mouse was fed a standard diet. The drinking bottle consisted of a 15-cm-long glass test tube fitted with a nozzle for precise measurements (Shinfactory, Fukuoka, Japan).

The sucrose preference test was also performed in single-housed mice. Each mouse was offered a drinking bottle containing tap water or a solution containing either 1% or 10% (w/v) sucrose. After 24 h, the amount of solution consumed and the animal’s body weight were measured, and intake was calculated as described above. During the test, each mouse was fed a standard diet.

For measuring long-term sucrose consumption, each single-housed mouse was offered either tap water or a 30% (w/v) sucrose solution. During the 2-week test, each mouse was fed a standard diet, and the amounts of solution and food consumed, as well as body weight, were measured every 24 h.

The consumption of each diet was measured for single-housed mice. After fasting for 24 h, each mouse was fed the LSD, HSD, LFD, or HFD. After 2, 4, and 24 h, the amount of food consumed and the animal’s body weight were measured. During the test, each mouse was housed on bedding paper (Oriental Yeast), which allowed the researcher to retrieve any spilled food. Food intake was calculated as the ratio between the amount consumed (in kcal) and the animal’s body weight (in kg).

### PR task

These experiments were performed between 1 p.m. and 6 p.m. Before the behavioral test, the mice were acclimated to 10% (w/v) sucrose in tap water and to the operant chamber in order to prevent neophobia. During the behavioral test, the mice were food-restricted in order to maintain 85% of their free-feeding weight before the start of the analysis.

The experiments were performed in a model ENV-307W aluminum operant chamber (21.6 cm×17.8 cm×12.7 cm; Med Associates, Georgia, FL). Each chamber was equipped with a liquid receptacle and a switchable dipper (ENV-302RW, Med Associates) in the center of a front panel, and the liquid receptacle contained an infrared photocell beam (ENV-302HD, Med Associates) for detecting head entry into the receptacle. A retractable lever was mounted on each side of the receptacle, and a light stimulus was placed above each lever. The chamber was enclosed in a sound-attenuating box and illuminated with a house light during the trials. The apparatus was controlled by a computer program written in MED-PC language.

The training phase was composed of the following three continuous reinforcement schedules: FR-1, FR-2, and FR-3. One of the two levers was randomly assigned as the “active” lever, which triggered the delivery of a 20 μl of 10% (w/v) sucrose solution. During presentation of the levers, the lights above the levers were illuminated. During the FR-1 phase, a single press of the lever triggered delivery of the reward, after which the levers were retracted and the lights above the levers were switched off. Head entry into the liquid receptacle was detected as a break in the infrared photocell beam; 3 s after the beam was broken, the levers were presented again, and the lights above the levers were illuminated. Following two successive sessions of obtaining 50 or more rewards, the schedule was changed to FR-2, in which two active lever presses were required to trigger delivery of the reward. Training in the FR-2 schedule continued for three days, after which the schedule was changed to FR-3, which was continued for three more days. Each FR training round consisted of one session of 100 reward deliveries or 60 min (whichever occurred first). The number of rewards obtained during the session, the ratio between the number of inactive lever presses and the total number of lever presses, and the average latency between reward delivery and the subsequent head entry were measured. Scores on the final days of FR-1, FR-2, and FR-3 phases were reported.

After the FR-3 phase, the mice were subjected to the PR reinforcement schedule^31^. The PR session continued for three days. The response ratio schedule during the test was calculated using the formula ([5e^(*R* x 0.2)^] – 5), where *R* is the number of rewards obtained plus 1. Thus, the number of lever presses to obtain the reward followed the order: 1, 2, 4, 6, 9, 12, 15, 20, 25, 32, 40, and so on. Each PR session continued for a maximum of 60 min. A lack of lever presses in any 5-min period resulted in termination of the session. The final ratio completed was indicated as break point. Scores on the final day were reported.

### Open field test

The open field test was performed in an apparatus consisting of a gray floor (42 cm×42 cm) with 30-cm-high gray walls. The floor was illuminated at 170 lux and was divided into a center zone (the central 20 cm×20 cm) and a peripheral zone (the area outside of the center zone). Each mouse was placed in the center of the open field and allowed to explore freely for 60 min. A video tracking system (Viewer 2, Biobserve, Bonn, Germany) was used to measure the precise trajectory of each mouse. For each mouse, the total distance traveled, the time spent in the center zone, and the number of entries into the center zone were measured. The mice were subjected to the open field test for three consecutive days, and the responses in the novel environment (day 1) and the acclimated environment (days 2 and 3) were evaluated.

### In vivo microdialysis and quantification of DA

Prior to the microdialysis experiment, the mice were acclimated to either water or 10% (w/v) sucrose in an agar in order to prevent neophobia. The mice were deeply anesthetized using isoflurane (1–2%), and a guide cannula (AG-8, Eicom, Kyoto, Japan) was surgically implanted in the medial NAc in the left hemisphere. The rostrocaudal, mediolateral, and dorsoventral coordinates (in mm) relative to Bregma or the dura were as follows: 1.5, 0.8, and 2.8, respectively, based on the mouse brain atlas^70^. At least five days after surgery, a dialysis probe (A-I-8–01, Eicom) equipped with a microinjection tube was inserted through the guide cannula, and then perfused overnight with artificial cerebrospinal fluid (consisting of 148 mM NaCl, 4 mM KCl, 0.85 mM MgCl_2_, and 1.2 mM CaCl_2_) at a flow rate of 1.0 μl/min using a microinfusion pump (ESP-32, Eicom). The membrane length and outer diameter of the dialysis probe was 1 mm and 0.22 mm, respectively. During the overnight perfusion, water deprivation was initiated at approximately 1 a.m., and dialysate was collected at 10-min interval beginning at approximately 12 p.m. (i.e., approximately 11 h after the start of water deprivation) using a refrigerated fraction collector (EFC-82, Eicom). At approximately 1 p.m., the mice were presented with either water or 10% (w/v) sucrose in an agar for 30 min. After the agar was removed, the dialysates were continued to be collected for an additional 90 min. During the test, each mouse was fed a standard diet. After the microdialysis experiments, the mice were sacrificed and histological analyses using cresyl violet staining were performed in order to verify proper placement of the probe (Supplementary Fig. 8). We excluded mice from subsequent analyses when the probe tip was not in the medial NAc.

The concentration of DA in each dialysate was measured using a high-performance liquid chromatography system equipped with an electrochemical detector (ECD-300, Eicom) in accordance with the manufacturer’s instructions. Data were collected and analyzed by using the Power Chrom version 2.2 (eDAQ, New South Wales, Australia). The baseline and post-meal periods were measured as the fractions collected in the first 60 min and the final 30 min, respectively.

### Immunohistochemistry

Embryos and anesthetized pups were perfused transcardially with 4% (w/v) paraformaldehyde (PFA) in PBS. Adults were anesthetized and perfused transcardially with PBS followed by 4% (w/v) PFA in PBS. The brains were removed and placed in 4% (w/v) PFA in PBS overnight at 4 °C. The embryonic and postnatal brains were then equilibrated in 20% or 30% (w/v) sucrose in PBS, respectively, and then embedded in OCT compound. The embryonic and postnatal brains were then sectioned using a cryostat (Leica, Wetzlar, Germany) at 12 μm or 30 μm, respectively.

Immunostaining was performed as previously reported^20^, and the nuclei were counterstained with DAPI (D9542, Sigma-Aldrich, St. Louis, MO). The sections were scanned using a DM6000B fluorescence microscope (Leica), LSM 510 confocal microscope (Carl Zeiss, Thornwood, NY), or A1R confocal microscope (Nikon, Tokyo, Japan). Confocal images of CTB-Alexa Fluor 488 were gamma-corrected due to high background noise.

The following primary antibodies were used for these experiments: mouse monoclonal anti-TH IgG (1:1000; MAB318, Millipore, Billerica, MA), rabbit polyclonal anti-TH antibody (1:1000; AB152, Millipore), rabbit monoclonal anti-DBH IgG (1:1000; ab209487, Abcam, Cambridge, UK), and rabbit monoclonal anti-active Caspase 3 IgG (1:400; 559565, BD Biosciences, San Jose, CA). The following secondary antibodies were used: Alexa Fluor 488-conjugated goat anti-mouse IgG (1:400; A11029, Invitrogen, Carlsbad, CA), Alexa Fluor 488-conjugated goat anti-rabbit IgG (1:400; A11034, Invitrogen), Alexa Fluor 647-conjugated donkey anti-mouse IgG (1:400; A31571, Invitrogen), Cy3-conjugated donkey anti-mouse IgG (1:400; 715–165–150, Jackson ImmunoResearch, West Grove, PA), and Cy3-conjugated donkey anti-rabbit IgG (1:400; 711–165–152, Jackson ImmunoResearch). The combinations of antibodies used in this study are summarized in Supplementary Table 1.

### CTB retrograde tracing analysis

Mice were deeply anesthetized with isoflurane (1–2%), and cholera toxin B subunit (CTB) conjugated to Alexa Fluor 488 or Alexa Fluor 555 (Invitrogen) dissolved in PBS to a final concentration of 0.5 mg/ml was infused into the medial or lateral NAc, respectively, through a glass microinjection capillary connected to a microinfusion pump (Eicom). The rostrocaudal, mediolateral, and dorsoventral coordinates (in mm) relative to Bregma or the dura were as follows: 1.5, 0.8, and 4.0 (for the medial NAc) and 1.0, 1.8, and 3.92 (for the lateral NAc), based on the mouse brain atlas^70^. The solution was injected at a constant flow rate of 0.1 μl/min (0.1 μl total/site).

### Intrabrain infusion of DA receptor antagonist

Mice were deeply anesthetized with isoflurane (1–2%), and a bilateral 26-gauge guide cannula (Plastics One, Roanoke, VA) was surgically implanted in the medial NAc. The rostrocaudal, mediolateral, and dorsoventral coordinates (in mm) relative to Bregma or the dura were as follows: 1.5, ±0.75, and 2.4, respectively, based on the mouse brain atlas^70^. After surgery, the mice were single-housed and fed a standard diet. At least 7 days after surgery, the mice received two daily bilateral infusions of 0.25 μl saline in order to acclimate to the infusion procedure. On the days of the water or sucrose consumption test, the mice received a bilateral infusion of 0.25 μl saline or cis-(Z)-flupenthixol (F114, Sigma-Aldrich) dissolved in saline. A 33-gauge bilateral internal cannula (Plastic One) equipped with a microinjection tube was inserted into the implanted guide cannula, and the internal cannula extended an additional 1.0 mm into the brain. After infusion at a flow rate of 0.25 μl/min with a microinfusion pump (Eicom), the internal cannula was left in place for an additional minute in order to permit diffusion of the drug. After each infusion, the mice were kept in their home cage for an additional 10 min in order to allow the drug to take effect before the water or sucrose consumption test began. After the behavioral experiments, the mice were sacrificed and histological analyses using cresyl violet staining were performed in order to verify the cannula placement sites (Supplementary Fig. 9).

### Targeted LC/MS/MS-based lipidomics

To obtain the ventral midbrain, 1-mm-thick coronal sections were prepared using a brainslicer (BS-Z 2000C, Muromachi Kikai, Tokyo, Japan), and the ventral midbrain was manually dissected from the relevant sections based on the mouse brain atlas^70^.

The LC/MS/MS analysis was performed as described previously^71^. Samples were extracted by solid-phase extraction using Monospin C18 (GL Sciences, Tokyo, Japan) with deuterium-labeled internal standards (AEA-d8, 1-AG-d8, and 2-AG-d4; Cayman Chemical, Ann Arbor, MI). The targeted analysis was performed using a UPLC system (Waters, Milford, MA) with triple quadruple linear ion trap mass spectrometer (QTRAP 5500, Sciex, Tokyo, Japan), equipped with Acquity UPLC BEH C18 column (1.0 × 150 mm 1.7 μm particle size; Waters). Samples were eluted with a mobile phase composed of water/acetate (100:0.1, v/v) and acetonitrile/methanol (4:1, v/v) using a gradient program as described^71^. The MS/MS analyses were performed in positive ion mode, and lipids were identified and quantified using calibration curves by multiple reaction monitoring. Data were collected by using the SCIEX Analyst version 1.7 (Sciex) and analyzed by using the SCIEX MultiQuant version 3.0 (Sciex).

### EdU analysis

Pregnant mice received an intraperitoneal injection of EdU (A10044, Thermo Fisher, Waltham, MA) dissolved in saline at a dose of 50 mg/kg body weight. The offspring’s brains were collected and prepared for histological analysis as described above. EdU was labeled with Alexa Fluor 488 using a Click-iT Plus EdU Imaging Kit (C10637, Thermo Fisher) in accordance with the manufacturer’s instructions.

### Statistics and reproducibility

The statistical tests used for each experiment are summarized in Supplementary Data 1; all tests were two-sided. Differences with a *P*-value **<**0.05 were considered statistically significant, unless stated otherwise. Statistical data were analyzed by using the Excel Statistics version 2.1 (Social Survey Research Information, Tokyo, Japan) and the SPSS Statistics version 25 (IBM, Armonk, NY). All statistical and quantitative data for individual samples are shown in Supplementary Data 1 and Supplementary Data 2, respectively. The investigators were blinded to all the quantitative analyses as samples were coded using numbers. We did not use statistical methods to calculate sample sizes, because the magnitudes of the effect sizes were not previously known; however, our sample sizes are similar to those reported in previous publications in the field. We replicated all experiments at least three times with similar results. The numbers of samples shown in the legends indicate those of individual mice or diets used in the experiments, unless otherwise stated. Except where indicated otherwise, all summary data are presented as the mean ± SEM.

### Reporting summary

Further information on research design is available in the Nature Research Reporting Summary linked to this article.

## Supplementary information

Supplementary Information

Description of Additional Supplementary Files

Supplementary Data 1

Supplementary Data 2

Reporting Summary

## Data Availability

All source data underlying the graphs and charts are available in Supplementary Data 2. All other data (if any) are available upon reasonable request.
