# Supplementary material for: Maternal dietary imbalance between omega-6 and omega-3 fatty acids triggers the offspring’s overeating in mice
Source: Commun Biol. 2020 Aug 28;3:473. doi: 10.1038/s42003-020-01209-4 (PMC7455742; doi:10.1038/s42003-020-01209-4)
Supplement: Supplementary file 1 — Supplementary Information [file 42003_2020_1209_MOESM1_ESM.pdf]

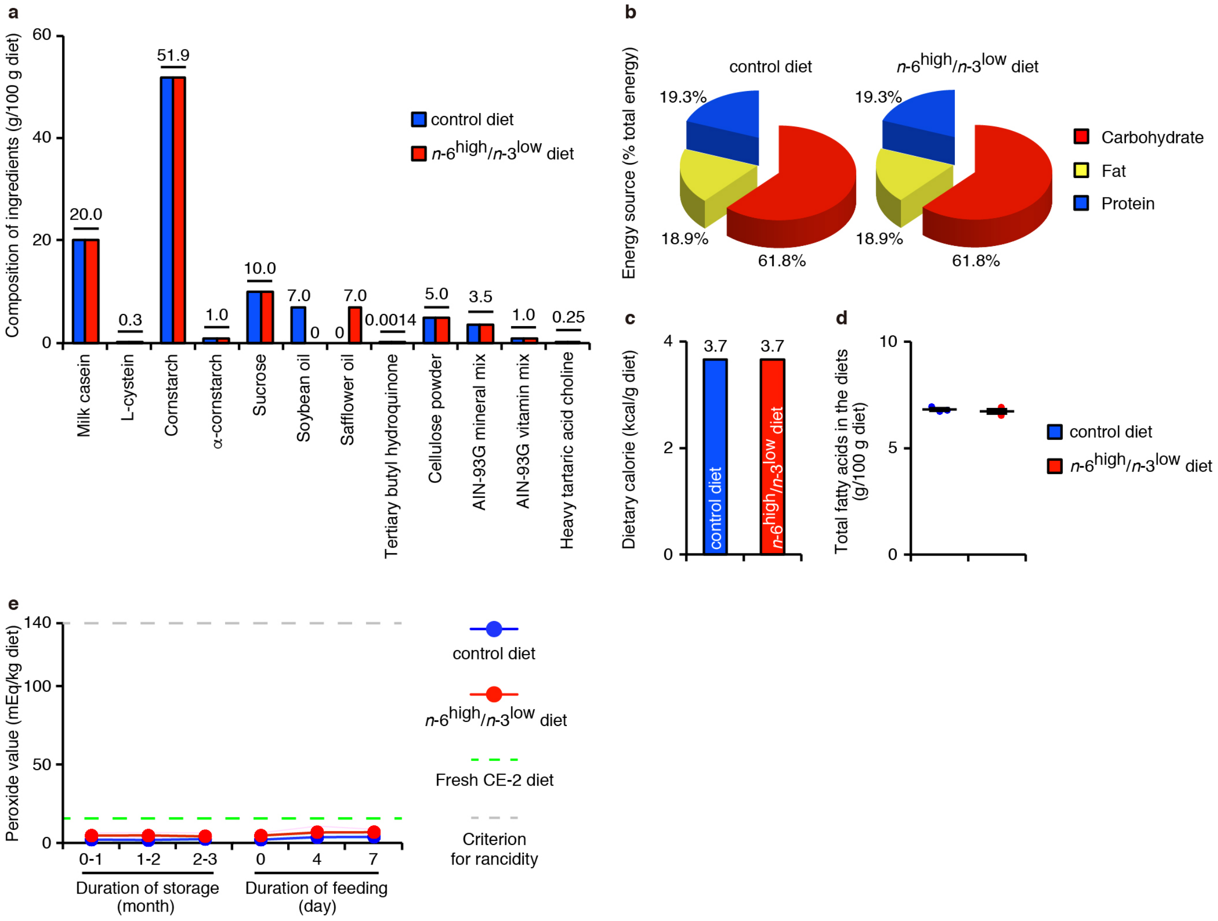

Supplementary Figure 1. Composition of the diets used in this study.

a-d, Composition, energy sources, caloric content, and total fatty acid content (n = 3/diet) in the control and n-6^{high}/n-3^{low} diets. Data were analyzed using an unpaired Student's t-test (**d**). **e**, Fatty acids in the diets do not undergo peroxidation during the experiments (n = 3/diet). For reference, the green and gray dashed lines show the peroxide values in freshly manufactured CE-2 diets and in standard deteriorated diets[1], respectively.

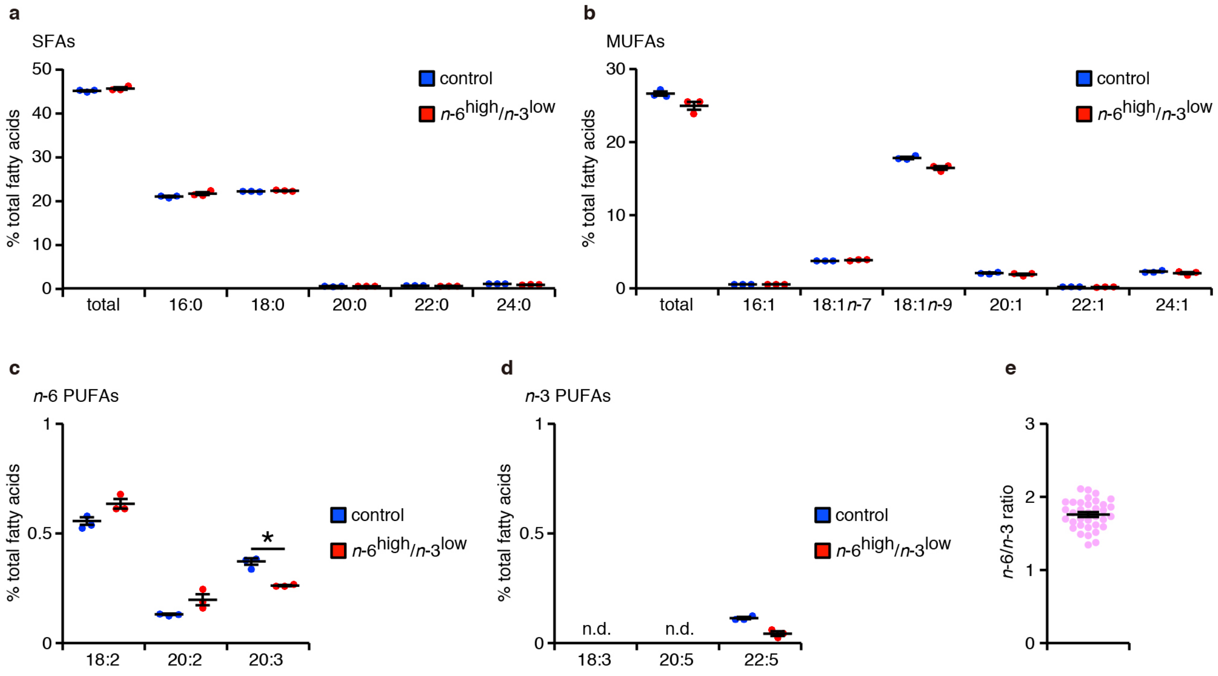

Supplementary Figure 2. Exposure to the *n*-6^{high}/*n*-3^{low} diet does not affect levels of saturated or monounsaturated fatty acids in the offspring's brain. **a-d**, Levels of saturated fatty acids (SFAs) (**a**), monounsaturated fatty acids (MUFAs) (**b**), minor *n*-6 PUFAs (**c**), and minor *n*-3 PUFAs (**d**) in the adult offspring's brain in the control and *n*-6^{high}/*n*-3^{low} groups (n = 3/group). $*P < 2.08 \times 10^{-3}$, unpaired Student's *t*-test. **e**, The *n*-6/*n*-3 ratio in the American postmortem brain (n = 35).

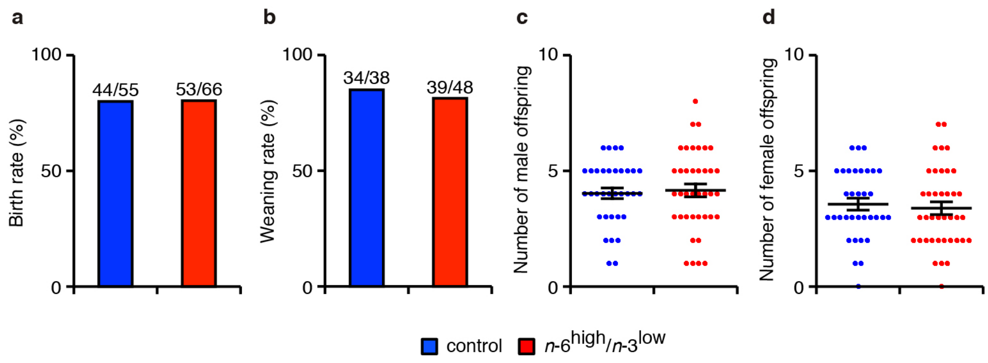

Supplementary Figure 3. Exposure to the *n*-6^{high}/*n*-3^{low} diet does not affect birth rate or postnatal mortality.

a-c, Birth rate (**a**), weaning rate (**b**), and the number of offspring per litter (**c,d**) measured in the control and *n*-6^{high}/*n*-3^{low} groups. Data were analyzed using a Pearson's χ^2 test (**a,b**), a Wilcoxon's rank sum test (**c**), or an unpaired Student's *t*-test (**d**). Data are expressed as the rate (**a,b**).

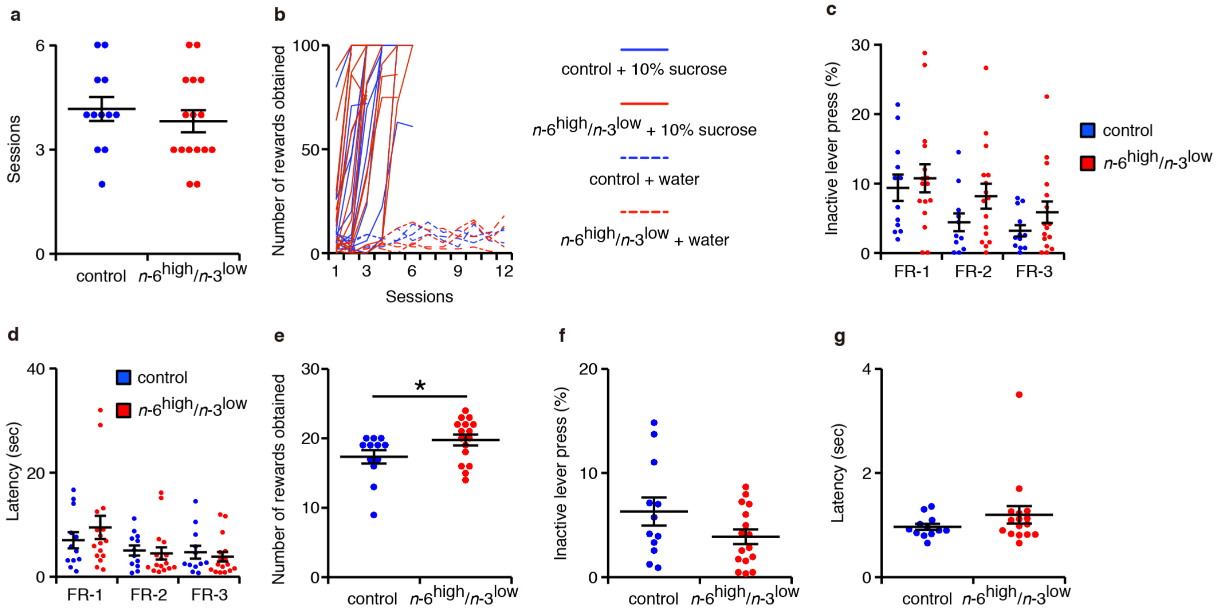

Supplementary Figure 4. The effect of exposure to the *n*-6^{high}/*n*-3^{low} diet on motivated behavior.

a, The number of sessions required to achieve ≥50 rewards in two successive sessions in the FR-1 task (*n* = 12/control or 16/*n*-6^{high}/*n*-3^{low}). Data were analyzed using an unpaired Student's *t*-test. **b**, The number of rewards obtained during the FR-1 task for each mouse in the control and *n*-6^{high}/*n*-3^{low} groups. Note that none of the mice reach the above-stated criterion when water was used as the reward (*n* = 3/group). **c,d**, The rate of pressing the inactive lever (**c**) and the latency to obtaining the reward (**d**) during the FR-1, FR-2, and FR-3 tasks (*n* = 12/control or 16/*n*-6^{high}/*n*-3^{low}). Data were analyzed using an unpaired Student's *t*-test (**c**, FR-1 and FR-2) or a Wilcoxon's rank sum test (**c**, FR-3; **d**, FR-1, FR-2, and FR-3)**. e-g**, The number of rewards obtained (**e**), the rate of pressing the inactive lever (**f**), and the latency to obtaining the reward (**g**) during the PR task (*n* = 12/control or 16/*n*-6^{high}/*n*-3^{low}). **P* < 0.05, Wilcoxon's rank sum test (**e,g**) or unpaired Student's *t*-test (**f**).

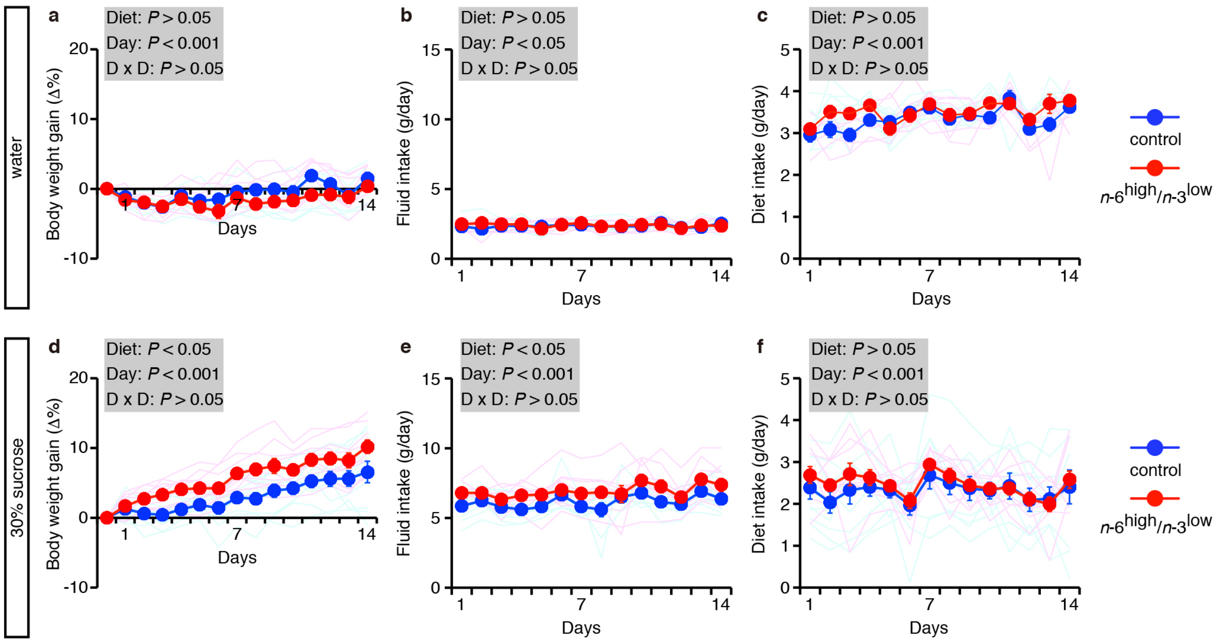

Supplementary Figure 5. The effect of exposure to the *n*-6$^{\mathbf{high}}$/*n*-3$^{\mathbf{low}}$ diet on long-term sucrose consumption.

a-f, Body weight gain (**a,d**), fluid intake (**b,e**), and food intake (**c,f**) measured in the control and *n*-6^{high}/*n*-3^{low} groups provided with water (n = 8/group) (**a-c**) or 30% sucrose solution (n = 10/group) (**d-f**). Data were analyzed using a two-way ANOVA (day as repeated measure).

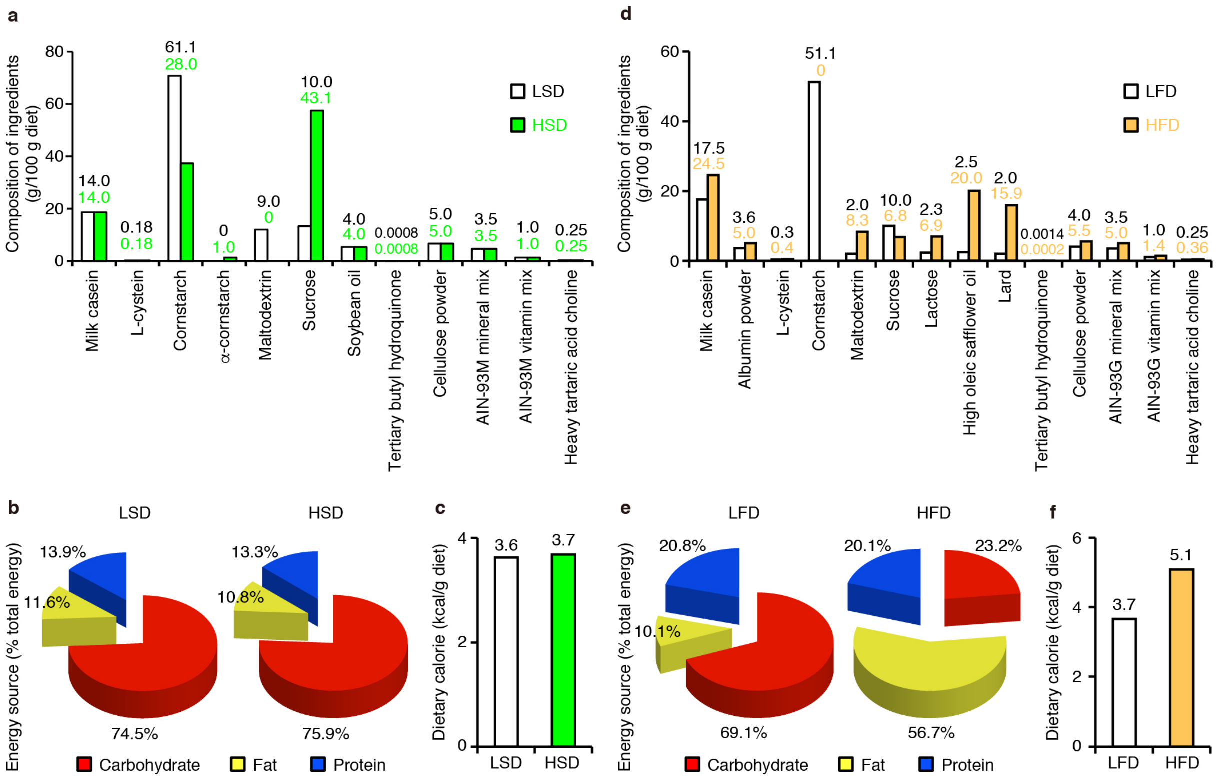

Supplementary Figure 6. Composition of highly palatable diets used in this study.

a,d, Composition of the LSD, HSD, LFD, and HFD. **b,e**, Energy sources of the indicated diets. **c,f**, Caloric content of the indicated diets.

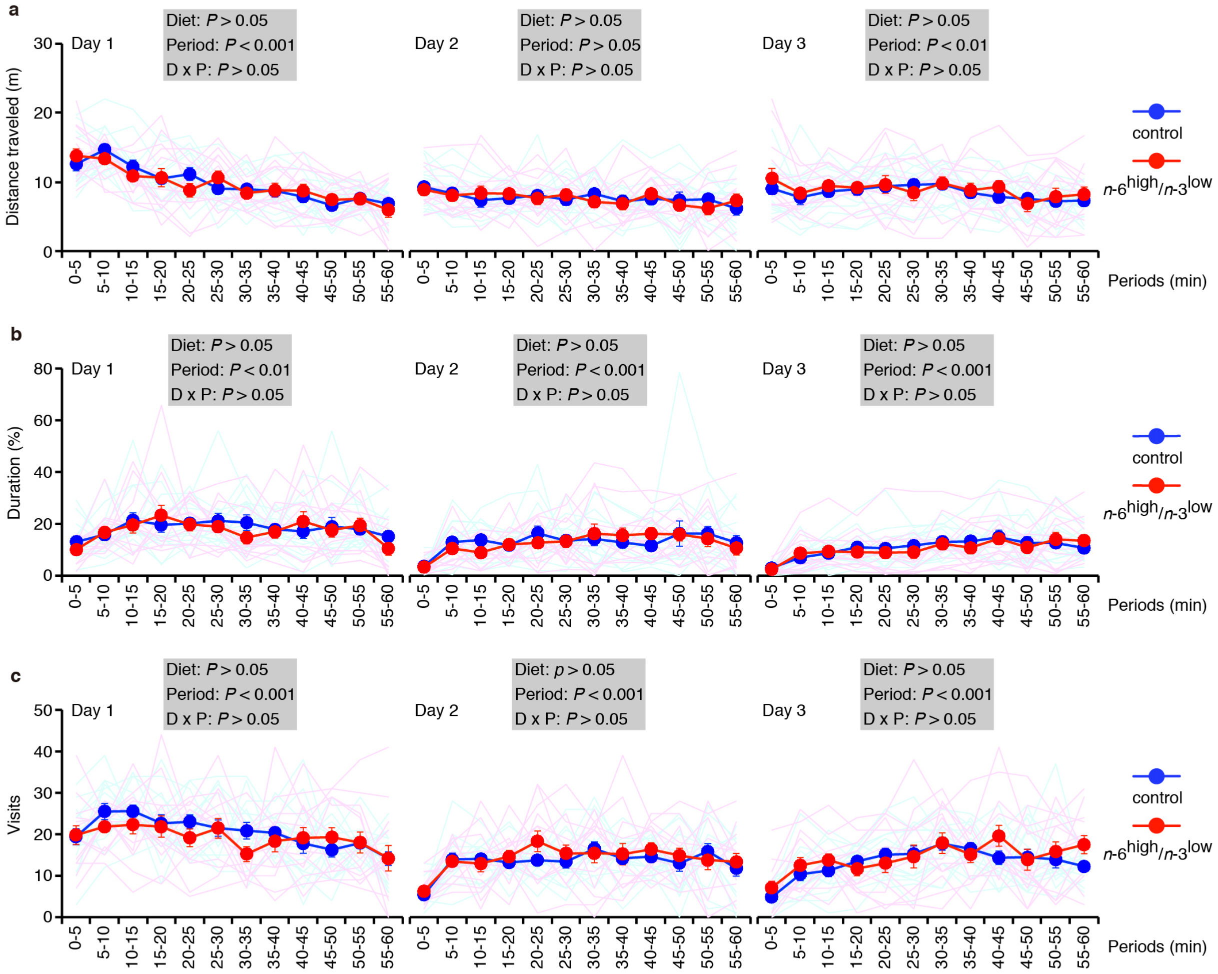

Supplementary Figure 7. The effect of exposure to the *n*-6^{high}/*n*-3^{low} diet on general behavior.

a-c, Distance traveled (**a**), time spent in the center zone (**b**), and the number of entries into the center zone (**c**) measured in the open field test for three consecutive days (n = 15/control or 14/*n*-6^{high}/*n*-3^{low}). Data were analyzed using a two-way ANOVA (period as repeated measure).

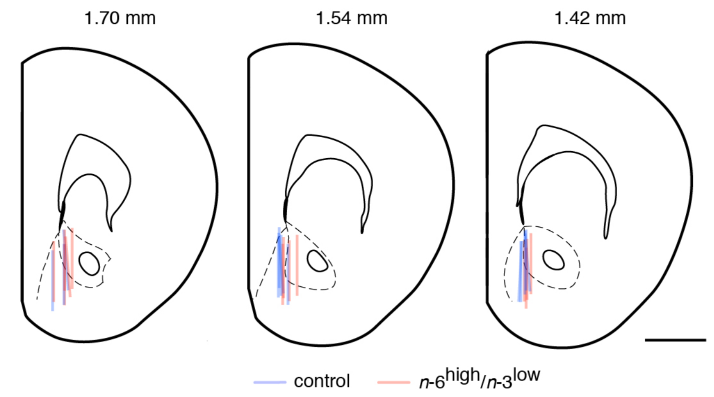

Supplementary Figure 8. Probe placement in the microdialysis experiments. Schematic drawings showing the position of the microdialysis probes in a coronal plane relative to Bregma. Scale bar = 1 mm.

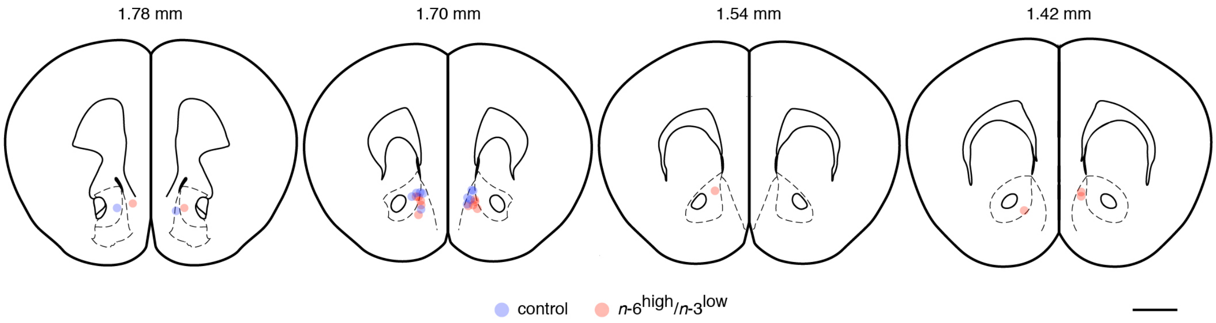

Supplementary Figure 9. Cannula placement in the flupenthixol infusion experiments.

Schematic drawings showing the tip placement of the infusion cannula in a coronal plane relative to Bregma. Scale bar = 1 mm.

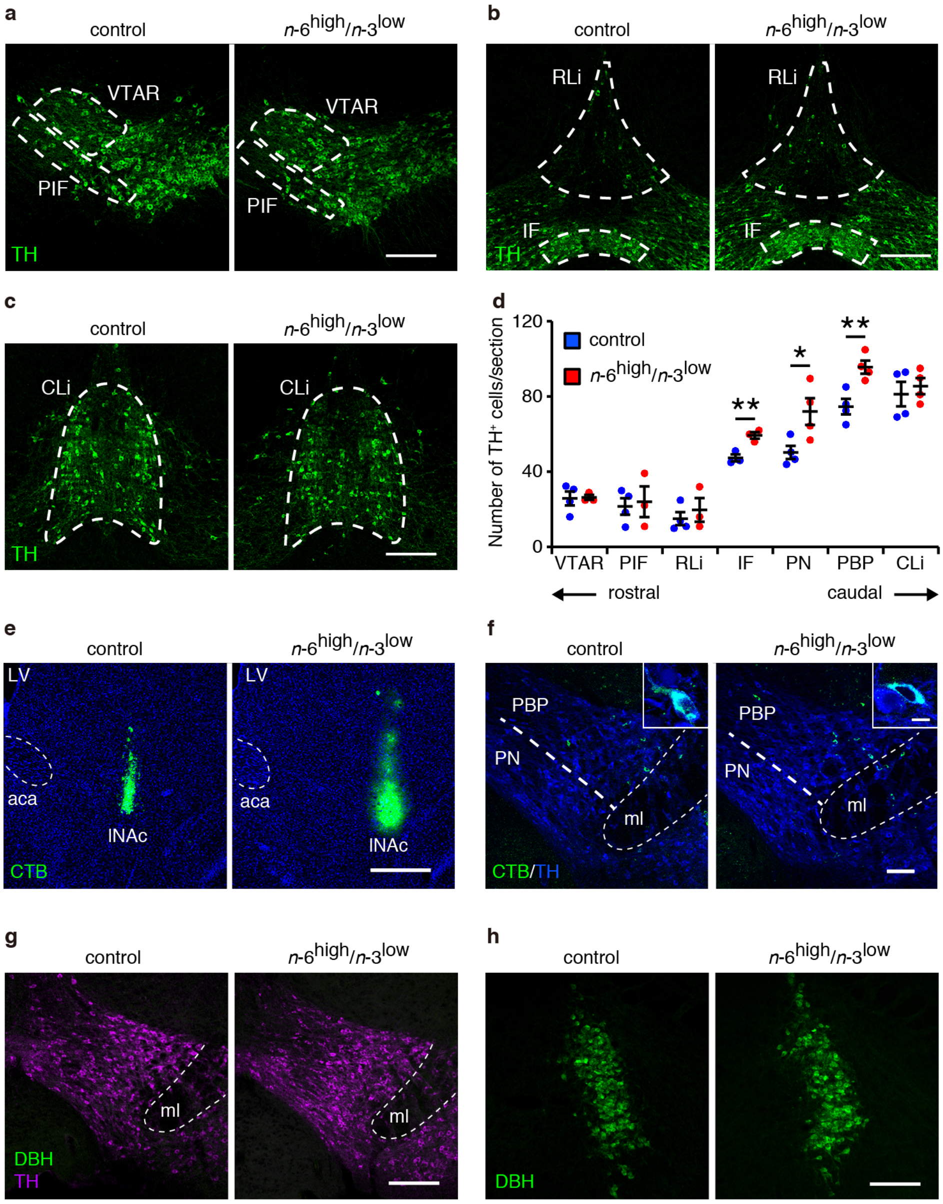

Supplementary Figure 10. Exposure to the *n*-6^{high}/*n*-3^{low} diet increases the number of dopaminergic neurons in the VTA.

a-d, TH-positive dopaminergic neurons measured from the rostral VTA to the caudal VTA (VTAR, PIF, and RLi, n = 4/control or 3/*n*-6^{high}/*n*-3^{low}; IF, n = 3/group; PN, PBP, and CLi, n = 4/group). *$P < 0.05$ and **$P < 0.01$, Wilcoxon's rank sum test (for the VTAR) or unpaired Student's *t*-test (for other subregions). **e**, Representative images of the CTB-Alexa Fluor 488 injection site in the lateral NAc (lNAc). The nuclei were counterstained with DAPI (blue). **f**, Dopaminergic

neurons innervating the lNAc is observed in the lPBP. **g**, DBH is not expressed in the VTA. **h**, As a control, we confirmed that DBH is expressed in the locus coeruleus. Scale bars = 200 µm (**a-c**,**g**,**h**), 500 µm (**e**), 100 µm (**f**), and 10 µm (**f**, inset). aca, anterior commissure; LV, lateral ventricle; ml, medial lemniscus; PIF, parainterfascicular nucleus; RLi, rostral linear nucleus; VTAR, ventral tegmental area rostral part.

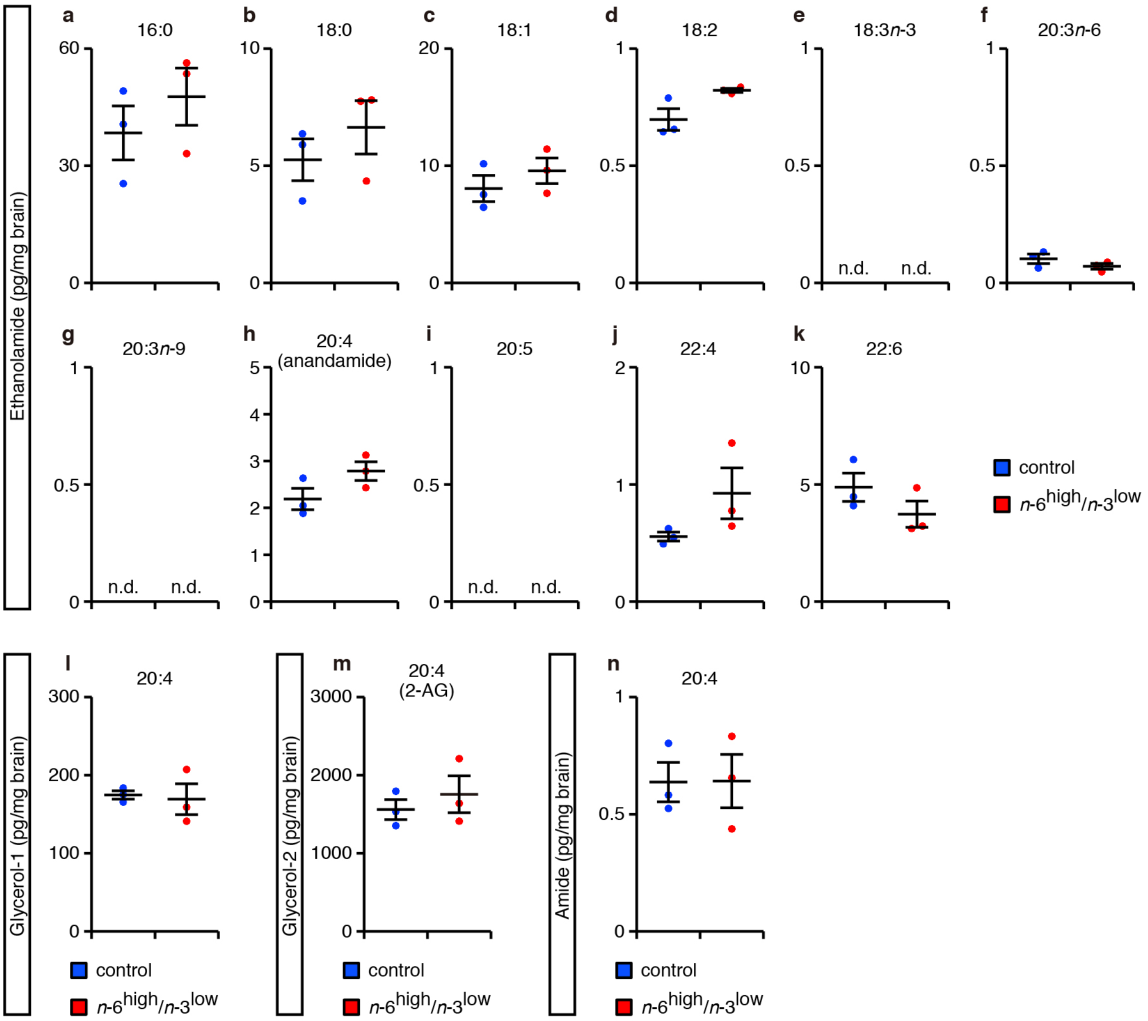

Supplementary Figure 11. The effect of exposure to the *n*-6^{high}/*n*-3^{low} diet on eCB biosynthesis.

a-n, The amounts of eCBs and related molecules measured in the ventral midbrain in the control and *n*-6^{high}/*n*-3^{low} groups (n = 3 samples/group). The ventral midbrains obtained from three mice were pooled and used as one sample. Data were analyzed using a Wilcoxon's rank sum test (**b**) or an unpaired Student's *t*-test (**a,c-n**). 2-AG, 2-arachidonoylglycerol.

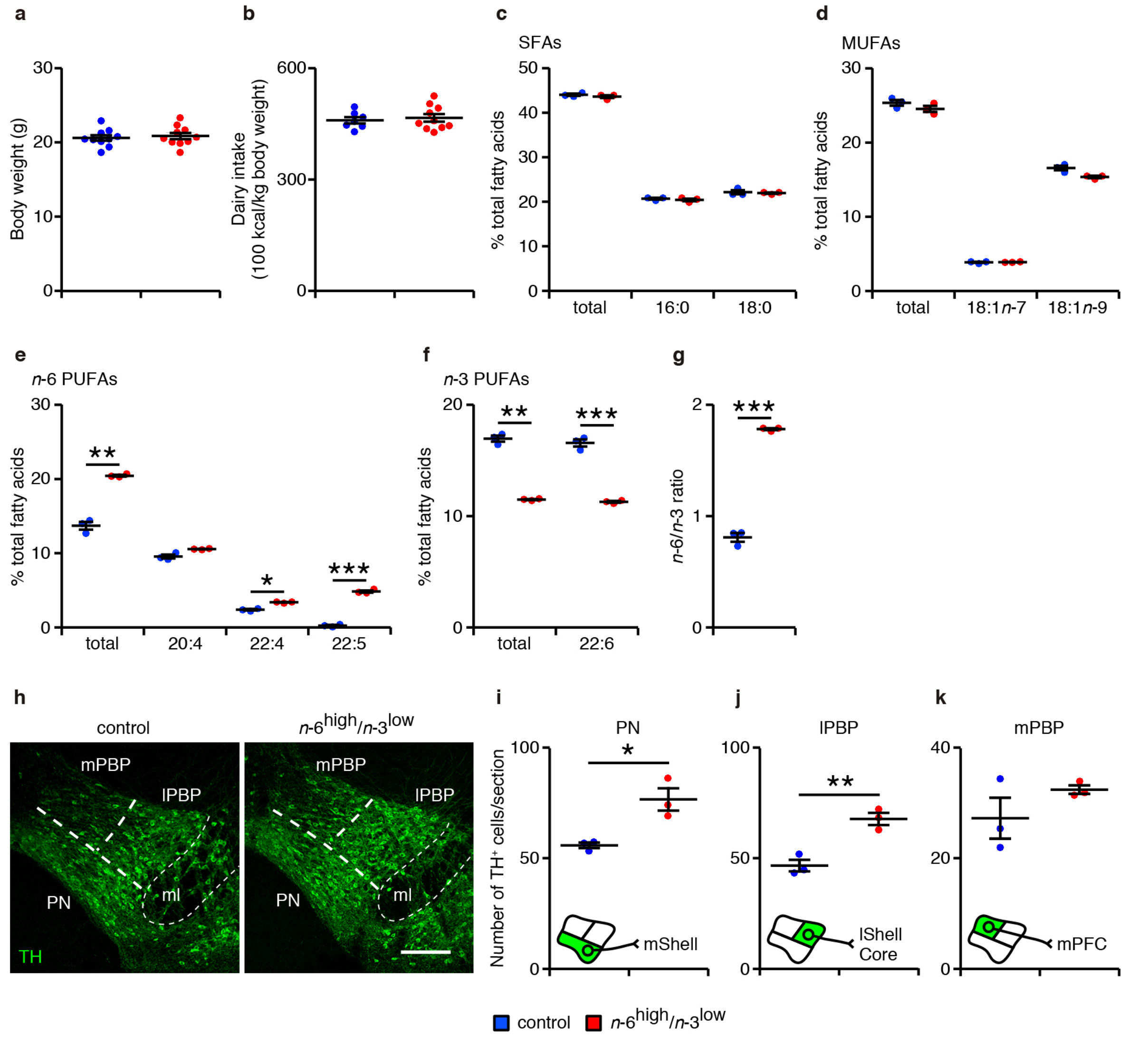

Supplementary Figure 12. The effect of exposure to the *n*-6^{high}/*n*-3^{low} diet on female offspring.

a, Body weight measured in the adult female offspring in the control and *n*-6^{high}/*n*-3^{low} groups (n = 10/group). Data were analyzed using an unpaired Student's t-test. **b**, Daily food intake measured in the adult female offspring in the control and *n*-6^{high}/*n*-3^{low} groups (n = 7/control or 10/*n*-6^{high}/*n*-3^{low}). Data were analyzed using an unpaired Student's t-test. **c-f**, Levels of major saturated fatty acids (SFAs) (**c**), monounsaturated fatty acids (MUFAs) (**d**), *n*-6 PUFAs (**e**), and *n*-3 PUFAs (**f**) in the adult female offspring brain in the control and

n-6^{high}/*n*-3^{low} groups (*n* = 3/group). *$P < 3.85 \times 10^{-3}$, **$P < 7.69 \times 10^{-4}$, ***$P < 7.69 \times 10^{-5}$, unpaired Student's *t*-test. **g**, The *n*-6/*n*-3 ratio in the adult female offspring brain in the control and *n*-6^{high}/*n*-3^{low} groups (*n* = 3/group). ***$P < 7.69 \times 10^{-5}$, unpaired Student's *t*-test. **h-k**, TH-positive dopaminergic neurons measured in the PN, lPBP, and mPBP in the control and *n*-6^{high}/*n*-3^{low} groups (*n* = 3/group). *$P < 0.05$, **$P < 0.01$, unpaired Student's *t*-test. Scale bar = 200 μm.

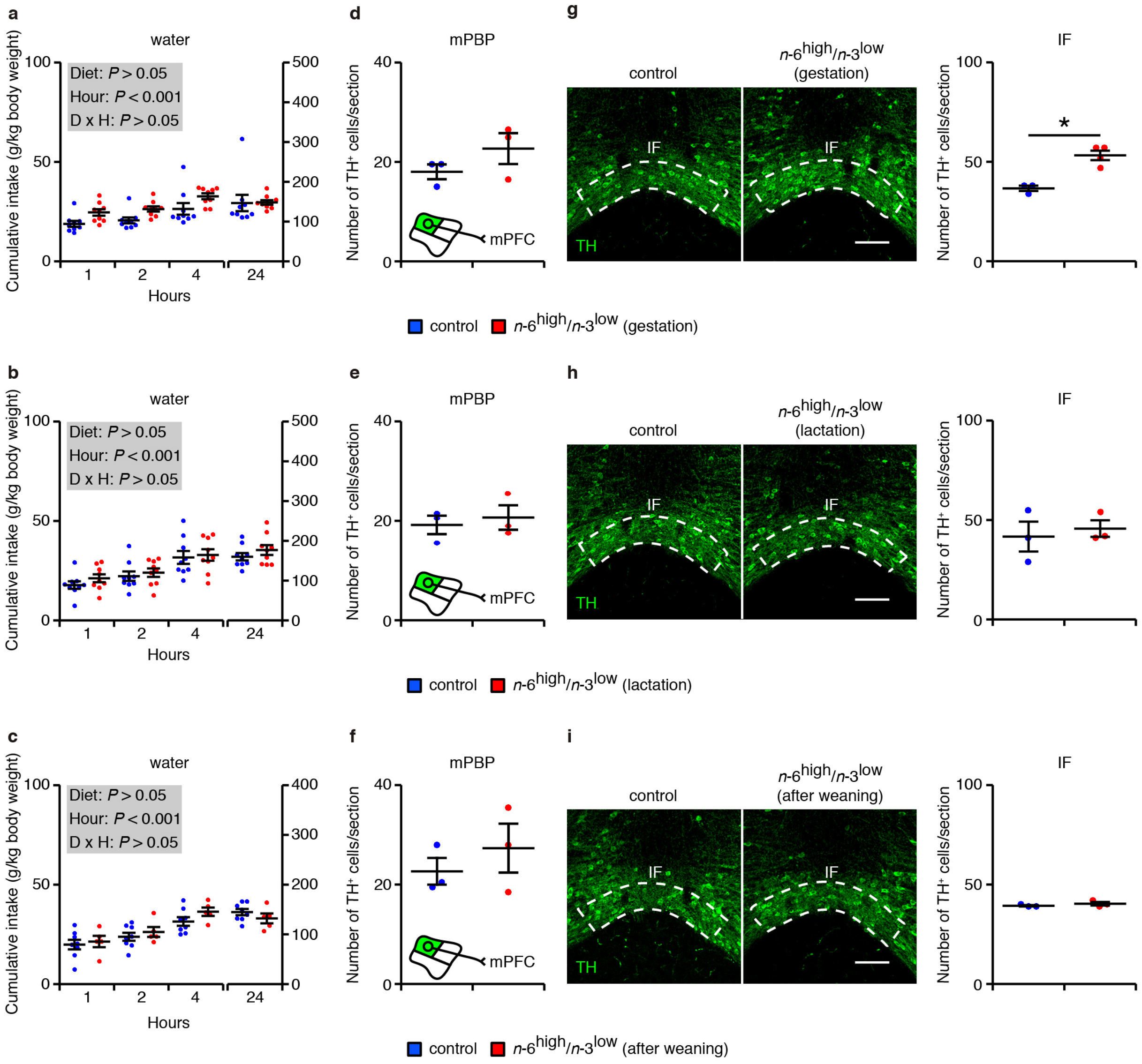

Supplementary Figure 13. The effect of exposure to the n-6^{high}/n-3^{low} at specific periods on water intake and the number of dopaminergic neurons in the VTA.

a-c, Cumulative water intake measured in the control group and in mice exposed to the n-6^{high}/n-3^{low} diet during gestation (**a**), during lactation (**b**), or after weaning (**c**) following 12 hours of water deprivation (**a,b**, n = 9/group; **c**, n = 8/control or 5/n-6^{high}/n-3^{low}). Data were analyzed using a two-way ANOVA (hour as repeated measure). **d-f**, TH-positive dopaminergic neurons measured in the mPBP (n = 3/group). Data were analyzed using a Wilcoxon's rank sum test (**d**) or

an unpaired Student's *t*-test (**e,f**). **g-i**, TH-positive dopaminergic neurons measured in the IF (n = 3/group). *$P < 0.05$, Wilcoxon's rank sum test (**g,i**) or unpaired Student's *t*-test (**h**). Scale bars = 100 μm. mPFC, medial prefrontal cortex.

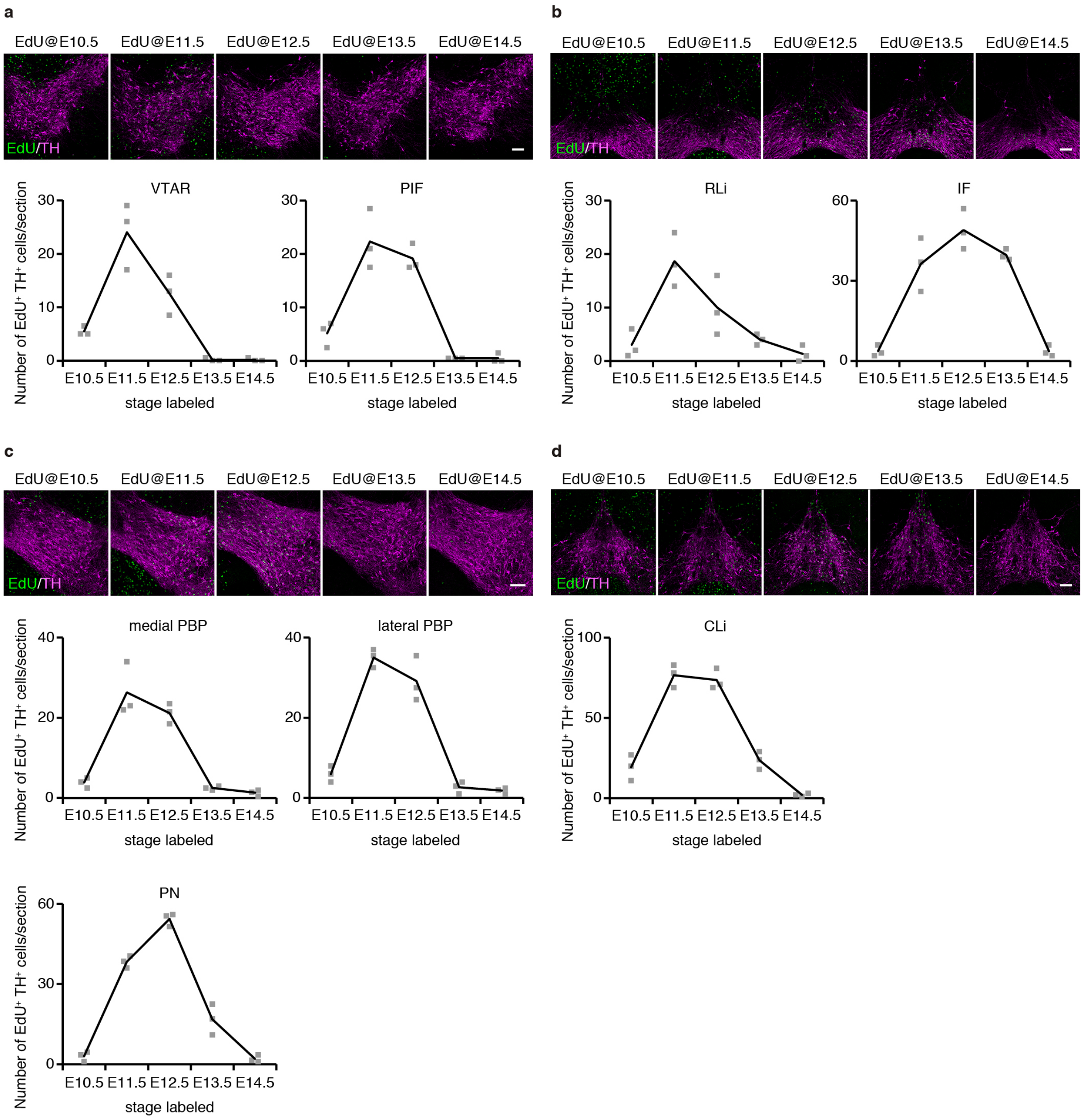

Supplementary Figure 14. Spatiotemporal pattern of dopaminergic neurogenesis in the developing VTA.

a-d, Pregnant mice were injected with EdU at the indicated gestational ages, and labeled dopaminergic neurons were measured in the offspring's VTA at P7 (n = 3/group). Scale bars = 100 μm. PIF, parainterfascicular nucleus; RLi, rostral linear nucleus; VTAR, ventral tegmental area rostral part.

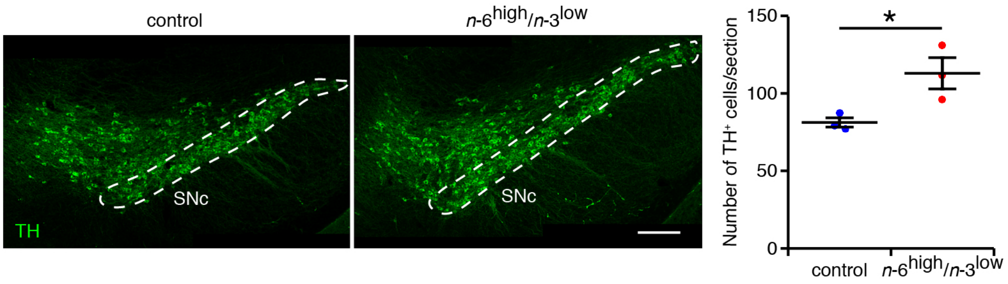

Supplementary Figure 15. Exposure to the *n*-6^{high}/*n*-3^{low} diet increases the number of dopaminergic neurons in the SNc.

TH-positive dopaminergic neurons measured in the SNc of adult mice in the control or *n*-6^{high}/*n*-3^{low} groups (n = 3/group). *$P < 0.05$, unpaired Student's *t*-test. Scale bar = 200 μm.

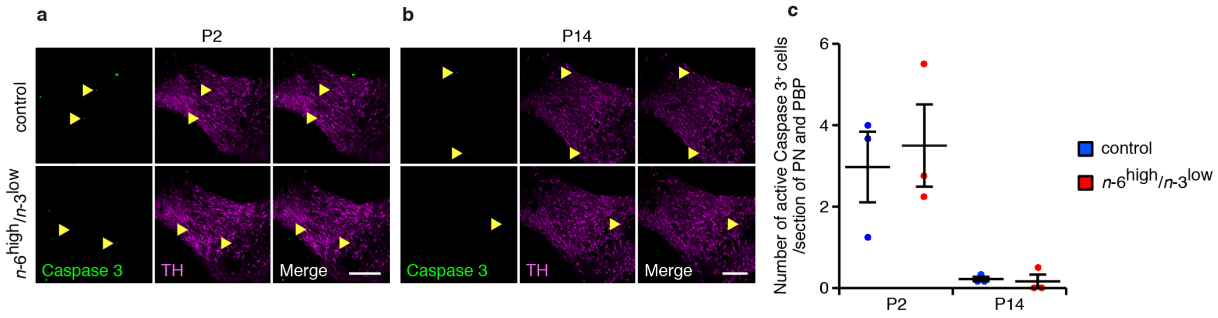

Supplementary Figure 16. Exposure to the *n*-6^{high}/*n*-3^{low} diet does not affect postnatal apoptosis in the developing VTA.

a-c, Active Caspase 3-positive cells in the PN and PBP measured in the control and *n*-6^{high}/*n*-3^{low} groups at P2 and P14 (n = 3/group). Data were analyzed using an unpaired Student's *t*-test (P2) or a Wilcoxon's rank sum test (P14). Scale bars = 200 μm. Arrowheads show active Caspase 3-positive cells in the PN and PBP.

Figure	Primary antibody	Secondary antibody
Figure 4a	mouse monoclonal anti-TH IgG (Millipore) 1:1000	Alexa Fluor 488-conjugated goat anti-mouse IgG (Invitrogen) 1:400
Figure 4g	mouse monoclonal anti-TH IgG (Millipore) 1:1000	Alexa Fluor 647-conjugated donkey anti-mouse IgG (Invitrogen) 1:400
Figure 5e-g	mouse monoclonal anti-TH IgG (Millipore) 1:1000	Alexa Fluor 488-conjugated goat anti-mouse IgG (Invitrogen) 1:400
Figure 6a-c	rabbit polyclonal anti-TH antibody (Millipore) 1:1000	Alexa Fluor 488-conjugated goat anti-rabbit IgG (Invitrogen) 1:400
Figure 6e,f	rabbit polyclonal anti-TH antibody (Millipore) 1:1000	Cy3-conjugated donkey anti-rabbit IgG (Jackson ImmunoResearch) 1:400
Supplementary Figure 10a-c	mouse monoclonal anti-TH IgG (Millipore) 1:1000	Alexa Fluor 488-conjugated goat anti-mouse IgG (Invitrogen) 1:400
Supplementary Figure 10f	mouse monoclonal anti-TH IgG (Millipore) 1:1000	Alexa Fluor 647-conjugated donkey anti-mouse IgG (Invitrogen) 1:400
Supplementary Figure 10g	rabbit monoclonal anti-DBH antibody (Abcam) 1:1000	Alexa Fluor 488-conjugated goat anti-rabbit IgG (Invitrogen) 1:400
	mouse monoclonal anti-TH IgG (Millipore) 1:1000	Cy3-conjugated donkey anti-mouse IgG (Jackson ImmunoResearch) 1:400
Supplementary Figure 10h	rabbit monoclonal anti-DBH antibody (Abcam) 1:1000	Alexa Fluor 488-conjugated goat anti-rabbit IgG (Invitrogen) 1:400
Supplementary Figure 12h	rabbit polyclonal anti-TH antibody (Millipore) 1:1000	Alexa Fluor 488-conjugated goat anti-rabbit IgG (Invitrogen) 1:400
Supplementary Figure 13g-i	mouse monoclonal anti-TH IgG (Millipore) 1:1000	Alexa Fluor 488-conjugated goat anti-mouse IgG (Invitrogen) 1:400
Supplementary Figure 14a-d	rabbit polyclonal anti-TH antibody (Millipore) 1:1000	Cy3-conjugated donkey anti-rabbit IgG (Jackson ImmunoResearch) 1:400
Supplementary Figure 15	mouse monoclonal anti-TH IgG (Millipore) 1:1000	Alexa Fluor 488-conjugated goat anti-mouse IgG (Invitrogen) 1:400
Supplementary Figure 16a,b	rabbit monoclonal anti-active Caspase 3 IgG (BD Biosciences) 1:400	Alexa Fluor 488-conjugated goat anti-rabbit IgG (Invitrogen) 1:400
	mouse monoclonal anti-TH IgG (Millipore) 1:1000	Cy3-conjugated donkey anti-mouse IgG (Jackson ImmunoResearch) 1:400

Supplementary Table 1. The combinations of antibodies.

Supplementary References

1. Fullerton, F. R., Greenman, D. L. & Kendall, D. C. Effects of storage conditions on nutritional qualities of semipurified (AIN-76) and natural ingredient (NIH-07) diets. *J Nutr* **112**, 567-573 (1982).
