# Supplementary material for: Maternal dietary imbalance between omega-6 and omega-3 fatty acids triggers the offspring’s overeating in mice
Source: Commun Biol. 2020 Aug 28;3:473. doi: 10.1038/s42003-020-01209-4 (PMC7455742; doi:10.1038/s42003-020-01209-4)
Supplement: Supplementary file 2 — Description of Additional Supplementary Files [file 42003_2020_1209_MOESM2_ESM.pdf]

Description of Additional Supplementary Files

File Name: **Supplementary Data 1**
Description: All statistical values in this study.

File Name: **Supplementary Data 2**
Description: All quantitative data for individual samples.
