# Supplementary material for: Maternal dietary imbalance between omega-6 and omega-3 fatty acids triggers the offspring’s overeating in mice
Source: Commun Biol. 2020 Aug 28;3:473. doi: 10.1038/s42003-020-01209-4 (PMC7455742; doi:10.1038/s42003-020-01209-4)
Supplement: Supplementary file 5 — Reporting Summary [file 42003_2020_1209_MOESM5_ESM.pdf]

nature research

Corresponding author(s): Nobuyuki Sakayori

Last updated by author(s): July 13, 2020

Reporting Summary

Nature Research wishes to improve the reproducibility of the work that we publish. This form provides structure for consistency and transparency in reporting. For further information on Nature Research policies, see Authors & Referees and the Editorial Policy Checklist.

Statistics

For all statistical analyses, confirm that the following items are present in the figure legend, table legend, main text, or Methods section.

n/a	Confirmed	
☐	☒	The exact sample size (*n*) for each experimental group/condition, given as a discrete number and unit of measurement
☐	☒	A statement on whether measurements were taken from distinct samples or whether the same sample was measured repeatedly
☐	☒	The statistical test(s) used AND whether they are one- or two-sided<br>*Only common tests should be described solely by name; describe more complex techniques in the Methods section.*
☐	☒	A description of all covariates tested
☐	☒	A description of any assumptions or corrections, such as tests of normality and adjustment for multiple comparisons
☐	☒	A full description of the statistical parameters including central tendency (e.g. means) or other basic estimates (e.g. regression coefficient) AND variation (e.g. standard deviation) or associated estimates of uncertainty (e.g. confidence intervals)
☐	☒	For null hypothesis testing, the test statistic (e.g. *F*, *t*, *r*) with confidence intervals, effect sizes, degrees of freedom and *P* value noted<br>*Give P values as exact values whenever suitable.*
☒	☐	For Bayesian analysis, information on the choice of priors and Markov chain Monte Carlo settings
☒	☐	For hierarchical and complex designs, identification of the appropriate level for tests and full reporting of outcomes
☒	☐	Estimates of effect sizes (e.g. Cohen's *d*, Pearson's *r*), indicating how they were calculated

Our web collection on statistics for biologists contains articles on many of the points above.

Software and code

Policy information about availability of computer code

Data collection	Data on the brain fatty acids were collected by using the Smart Chrom version 2.27J. Data on the brain dopamine were collected by using the Power Chrom version 2.2. Data on the brain endocannabinoids were collected by using the SCIEX Analyst version 1.7.
Data analysis	Data on the brain fatty acids were analyzed by using the Smart Chrom version 2.27J. Behavioral data in the open field test were analyzed by using the Viewer 2. Data on the brain dopamine were analyzed by using the Power Chrom version 2.2. Data on the brain endocannabinoids were analyzed by using the SCIEX MultiQuant version 3.0. Statistical data were analyzed by using the Excel Statistics version 2.1 and the SPSS Statistics version 25.

For manuscripts utilizing custom algorithms or software that are central to the research but not yet described in published literature, software must be made available to editors/reviewers. We strongly encourage code deposition in a community repository (e.g. GitHub). See the Nature Research guidelines for submitting code & software for further information.

Data

Policy information about availability of data

All manuscripts must include a data availability statement. This statement should provide the following information, where applicable:

- Accession codes, unique identifiers, or web links for publicly available datasets
- A list of figures that have associated raw data
- A description of any restrictions on data availability

All data generated during this study are available within the paper and its supplementary information files.

Field-specific reporting

Please select the one below that is the best fit for your research. If you are not sure, read the appropriate sections before making your selection.

☒ Life sciences ☐ Behavioural & social sciences ☐ Ecological, evolutionary & environmental sciences

For a reference copy of the document with all sections, see nature.com/documents/nr-reporting-summary-flat.pdf

Life sciences study design

All studies must disclose on these points even when the disclosure is negative.

Sample size	We did not use statistical methods to calculate sample sizes, because the magnitudes of the effect sizes were not previously known; however, our sample sizes are similar to those reported in previous publications in the field.
Data exclusions	For in vivo microdialysis, we excluded data when the probe tip was not in the medial NAc.
Replication	We replicated all experiments at least three times with similar results.
Randomization	Mice were randomly allocated to the dietary groups.
Blinding	The investigators were blinded to all quantitative analyses as samples were coded using numbers.

Reporting for specific materials, systems and methods

We require information from authors about some types of materials, experimental systems and methods used in many studies. Here, indicate whether each material, system or method listed is relevant to your study. If you are not sure if a list item applies to your research, read the appropriate section before selecting a response.

Materials & experimental systems

n/a	Involved in the study	
☐	☒	Antibodies
☒	☐	Eukaryotic cell lines
☒	☐	Palaeontology
☐	☒	Animals and other organisms
☒	☐	Human research participants
☒	☐	Clinical data

Methods

n/a	Involved in the study	
☒	☐	ChIP-seq
☒	☐	Flow cytometry
☒	☐	MRI-based neuroimaging

Antibodies

Antibodies used	mouse monoclonal anti-TH IgG (MAB318, Millipore); rabbit polyclonal anti-TH antibody (AB152, Millipore); rabbit monoclonal anti-DBH IgG (ab209487, Abcam); rabbit monoclonal anti-active Caspase 3 IgG (559565, BD Biosciences); Alexa Fluor 488-conjugated goat anti-mouse IgG (A11029, Invitrogen); Alexa Fluor 488-conjugated goat anti-rabbit IgG (A11034, Invitrogen); Alexa Fluor 647-conjugated donkey anti-mouse IgG (A31571, Invitrogen); Cy3-conjugated donkey anti-mouse IgG (715-165-150, Jackson ImmunoResearch); and Cy3-conjugated donkey anti-rabbit IgG (1:400; 711-165-152, Jackson ImmunoResearch)
Validation	All antibodies were validated by the company and by users.

Animals and other organisms

Policy information about studies involving animals; ARRIVE guidelines recommended for reporting animal research

Laboratory animals	We obtained C57BL/6J female mice from Clea Japan and used male and female offspring for the analyses. The age for each experiment is indicated in the manuscript.
Wild animals	n/a
Field-collected samples	n/a
Ethics oversight	All animal experiments were performed in accordance with the National Institutes of Health Guidelines for the Care and Use of Laboratory Animals and were approved by our university's committee for animal experimentation.

Note that full information on the approval of the study protocol must also be provided in the manuscript.
